# A deep neural network with attention mechanism for flow prediction of compressor blade

**DOI:** 10.1038/s41598-025-99688-0

**Published:** 2025-05-10

**Authors:** Guanyu Gao, Gang Wang

**Affiliations:** 1 State Key Lab of Reliability and Intelligence of Electrical Equipment, Hebei University of Technology, Tianjin, 300130 People’s Republic of China; 2 School of Mechanical Engineering, Hebei University of Technology, Tianjin, 300401 People’s Republic of China; 3 Tianjin Key Laboratory of Power Transmission and Safety Technology for New Energy Vehicles, Tianjin, 300130 People’s Republic of China

**Keywords:** Deep neural network, Flow prediction, Attention mechanism, Compressor blade, U-Net, Engineering, Mechanical engineering, Fluid dynamics

## Abstract

For flow-related design optimization problems, computational fluid dynamics (CFD) simulations are commonly used to predict the flow fields. However, the computational expenses of CFD simulations limit the opportunities for design exploration. Motivated by this tricky issue, a convolutional neural network (CNN) based on U-Net architecture with attention mechanism (AM) is proposed to efficiently learn flow representations from CFD results to shorten the compressor blade design cycle. The proposed model converts the provided shape information and flow conditions into grayscale images to directly predict the expected flow field, saving computational time. An extensive hyper-parameter search is performed to determine the optimal model. Qualitative and quantitative analysis of the results are studied to evaluate the accuracy for the calculation of Mach number distributions. In particular, two new attention mechanisms is developed to preserve the physical consistency of the complex flow field with shock wave. Mach number flow fields under different working conditions are predicted using the proposed model, and the prediction is well consistent with CFD results. Over three orders of magnitude of speedup is achieved at all batch sizes compared to traditional CFD methods, while maintaining low prediction errors.

## Introduction

With the increasing demands on load, performance and stability margin of compression system in modern aeroengines, fast and accurate determination of flow fields is critical for compressor design and optimization. Compressors manifest two distinct forms of hydrodynamic instability, namely surge and stall^1^. The occurrence of the above conditions is the stable boundary where the engine cannot operate safely. After determining the flow angle of each span, it is necessary to analyze compressor aerodynamic performance through flow field across different working conditions^2–4^. Therefore, the stable operating range of the compressor can be determined. The flow field of various spans serves as a cornerstone for investigating the efficacy of asymmetric end wall shapes in managing separated flow within the compressor, thereby enhancing the compressor’s aerodynamic performance and expanding its stability margins.

Scholars often employ CFD^5^ simulations to computationally tackle controlled Navier–Stokes equations^6^ with the aim of improving the flow field structure within the compressor cascade. Luo et al.^7^ established the relationship between topological structures and vortex structures in corner regions of tandem cascades, studying the flow losses and vortex structures. Zhang et al.^8^ used three suction schemes at different axial positions to study the effect of improving cascade performance and separating flow control. Cao et al.^9^ conducted hydrodynamic simulation for a tandem cascade based on an existing single-row cascade design, and the effects of the two designs were analyzed on various flow losses under identical working conditions. The aforementioned studies indicate that CFD can well simulate the flow field structure to study flow losses and separate flow control. Nevertheless, owing to the intricacies inherent in the physics underlying flow prediction, CFD solutions can entail substantial computational costs. This expense serves as a significant constraint in the context of aerodynamic design optimization^10,11^. This predicament is acute during the initial design phases, where engineers seek rapid assessments of the potential benefits associated with numerous design alternatives. Therefore, to achieve real-time analysis during aerodynamic design optimizations, there is an urgent need for developing an accurate and efficient method, which can obtain the flow field faster than the conventional CFD approach.

Neural networks, as a machine learning methodology, have garnered remarkable achievements in computer vision^12,13^, and when integrated with mechanics, they offer a potent approach for addressing challenges in physics and engineering^14–16^. In addition to the widely used areas mentioned above, their fusion with aerodynamics holds promise for resolving numerous issues in aerodynamic performance analysis^17–20^. Indeed, within aerodynamics, neural networks furnish a plethora of sophisticated models and algorithms capable of tackling diverse problems, encompassing the resolution of governing equations, aerodynamic forecasting and flow field reconstruction^21–23^. Neural networks can be used to address high-dimensional^24^, multi-scale^25^ and nonlinear problems^26^, which pose challenges for traditional CFD methods, and certain neural networks exhibit proficiency in processing time series data^27^. In the early stages of flow field reconstruction research, multi-layer perceptrons (MLPs) are used to reconstruct the steady flow field^18,28^, showcasing superiority over proper orthogonal decomposition (POD) reduced order models. Despite ensuring model stability, the accuracy of the generated data remained unsatisfactory. Subsequent investigations introduced CNNs, which could effectively capture the intricacies of turbulence modeling and reconstruct the velocity field in steady-state flow^29–31^. Using CNNs for flow field reconstruction not only guarantees the reliability of output data, but also captures the spatial distribution of steady-state flow field data. Furthermore, the CNN model has robust stability and can deal with sparse and noisy data effectively^32–35^, which ensures the feasibility and practicability of the data model. Ultimately, the model’s predictive capabilities can be enhanced by incorporating the physical information from mechanistic models, and aligning CNN model with the physical constraints^36–38^.

More recently, researchers have devote remarkable efforts into predicting the flow field using Deep Learning (DL). Portal-Porras et al.^39^ applied CNN to predict the flow behaviour on the wake behind Vortex Generators(VGs). The results show that the CNN can accurately predict the velocity and vorticity fields. Wang et al.^40^ developed a CNN model for the performance assessment of aeroengine turbines. The pressure and temperature fields are reconstructed through the model for multiple rotor profile conditions. Sun et al.^41^ investigated the effects of the input coordinate features of the deep neural network to predict compressible flows over the transonic airfoils. Lift, drag, and pitch coefficients can be obtained from the predicted flow fields. Overall, researchers have well applied DL in the prediction of flow fields, and using data-driven machine learning methods to yield accurate approximations of these simulations with a significantly reduced resource footprint holds great appeal .

Despite its promise, the field of CNN-based flow approximation remains in its nascent stage. Current methodologies only rely on statistical models of image processing, thereby exhibiting a fundamental flaw in current model lack of integration with practical engineering applications. Unlike photo images, flow field images represent visualizations of CFD numerical simulations, necessitating adherence to fundamental physical laws such as mass and momentum conservation. Certain recent studies are specifically tailored for modeling turbulence simulations, with model architectures intricately linked to specific simulation methods^42,43^. Consequently, there is currently a lack of generalizability to steady flow prediction and alternative simulation techniques. These approaches often yield substantial prediction errors in steady flow simulation scenarios. Previous studies have also overlooked the potential for optimization within CFD workloads to enhance prediction accuracy. Specifically, in flow field prediction, there’s a need to guide the model’s attention towards crucial regions such as boundary layers, as CFD simulations need to prioritize these intricate areas with sharp gradients. This differs from classical image processing tasks like object recognition, where attention is focused on target location and size. Existing CNN-based flow approximation methods merely adapt models developed for standard image processing, overlooking the nuances of CFD workloads and thus miss massive optimization opportunities.

In this work, a CNN-based deep neural network model with attention mechanisms is developed to provide an approximate solution to Mach number. The ground-truth data of internal flow field information generated by a mature turbomachinery CFD solver. The ground-truth data are inputted into the model to improve the quality and accuracy of flow prediction by taking advantage of the characteristics of CFD workloads. To capitalize on the unique features of CFD simulations, two new attention mechanisms are proposed to effectively extract features from regions with sharp gradients. The mean absolute error is less than 1% between prediction results and the CFD results while significantly improving solution speed by three orders of magnitude. The proposed model can predict the flow field structure within the compressor under various working conditions, providing a comprehensive solution for Mach number even with limited annotated data.

## Flow field prediction method

**Fig. 1:**
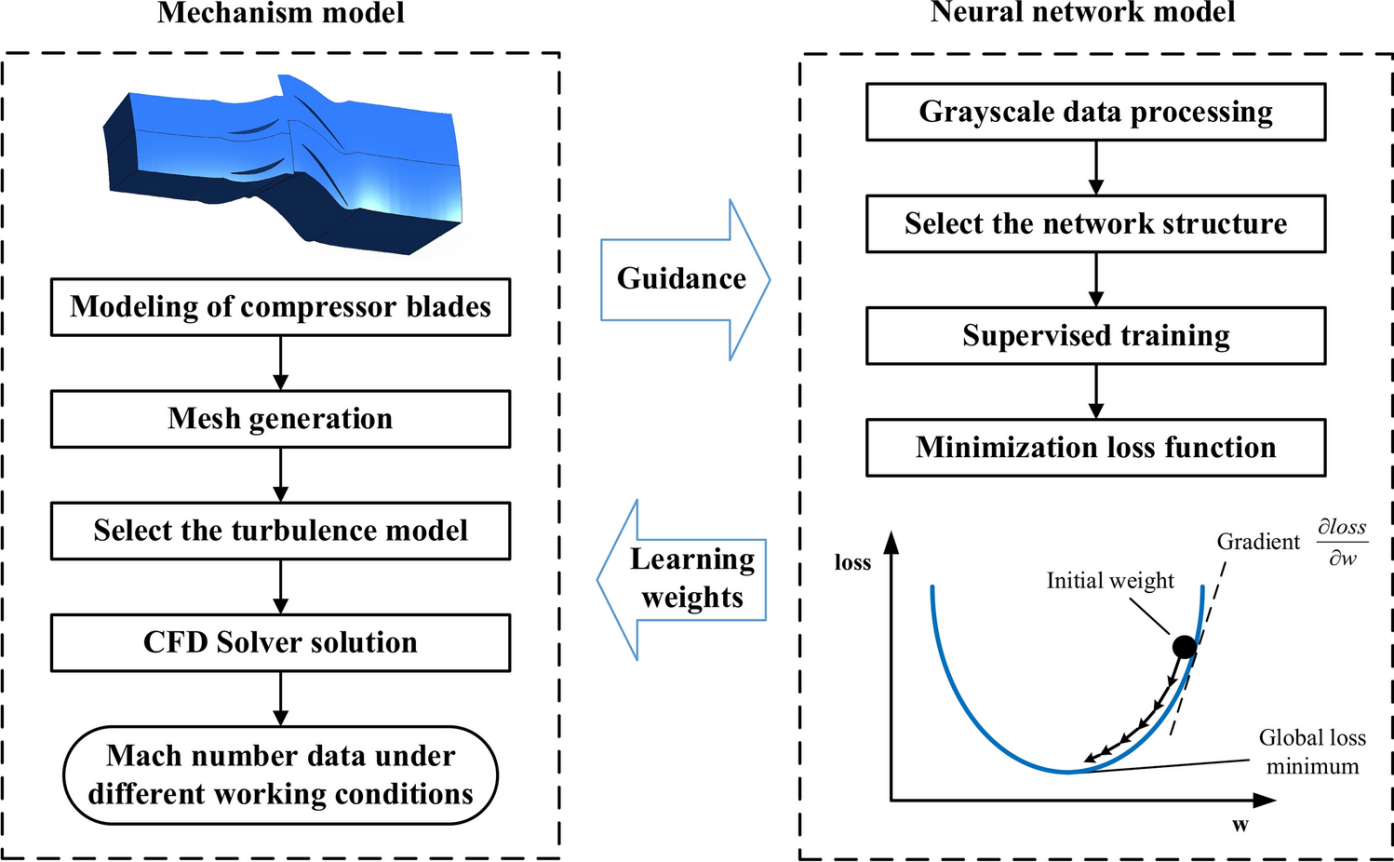
Steps to establish prediction model.

This section presents an introduction to traditional CFD method followed by the prediction model based on CNN. Traditional CFD methods serve as the source of ground-truth data for training the prediction model. CNN is employed to extract features from CFD results and predict the flow field of compressor blades. The prediction process is divided into two parts: data processing and supervised training. As shown in Fig. 1, the initial train data set is the Mach number field obtained by the CFD solver. For data processing, the shape information and the boundary conditions of the compressor blades are transformed into the grayscale image as the initial input of the train data set. For supervised training, the train data set is fed into CNN to optimize the net parameters. After training, the trained networks can be obtained.

### Flow field data collection

The governing equations of fluid mechanics include continuity equation, momentum equation and energy equation. The continuity equation has the form

1 $$\frac{\partial \rho }{{\partial t}} + \frac{{\partial \left( {\rho u_{i} } \right)}}{{\partial x_{i} }} = 0$$

where $\rho$ is the mass density, *x*_*i*_ is the displacement component, *u*_*i*_ is the velocity component.

The law of conservation of momentum describes the physical mechanism by which the time change of momentum is determined by the internal and external forces of the fluid. The momentum conservation equation of the fluid can be derived in the form of

2 $$\frac{{\partial (\rho u_{i} )}}{\partial t} + \frac{\partial }{{\partial x_{j} }}\left( {\rho u_{i} u_{j} } \right) = - \frac{\partial p}{{\partial x_{i} }} + \frac{\partial }{{\partial x_{j} }}\mu \left( {\frac{{\partial u_{i} }}{{\partial x_{j} }} + \frac{{\partial u_{j} }}{{\partial x_{i} }}} \right) + \lambda \frac{\partial }{{\partial x_{j} }}\left( {\mu \frac{{\partial u_{j} }}{{\partial x_{i} }}} \right)$$

where $\mu$ is the dynamic viscosity coefficient, *p* is static pressure. The formula includes inertia term, convection term, pressure gradient term, viscosity term and external force term.

The mathematical expression of energy conservation can be written as follows

3 $$\frac{\partial (\rho E)}{{\partial t}} + \frac{{\partial \left( {\rho Eu_{i} } \right)}}{{\partial x_{i} }} = F_{i} u_{j} + \frac{\partial }{{\partial x_{j} }}\left( {u_{i} \tau_{ij} } \right) - \frac{{\partial q_{i} }}{{\partial x_{j} }} + S_{E}$$

where *q*_*i*_ is the component of heat flux, *S*_*E*_ is the energy source term applied by the outside world to the fluid element, *E* is the total energy of the fluid element.

The research focuses on the NASA stage37 transonic stage as research object. The datasets for Mach number field prediction are obtained using the numerical simulation method, and the two-dimensional section of the Mach number fields are collected as the data of the input layer of each model. Following the determination of the turbulence model, grid-independent verification is conducted to establish the appropriate grid density. Reference to pertinent NASA experimental data enables comparison of total pressure ratio and efficiency variations with mass flow, verifying the reliability of numerical calculation. The key design parameters are detailed in Table 1^44^.

**Table 1:** Stage37 design parameters.

Parameters	Design value
Total pressure ratio	2.05
Rotative speed(rpm)	17,188.7
Mass flow(kg/s)	20.2
Total inlet pressure(Pa)	101,325
Total inlet temperature(K)	288.15
Inlet tip velocity(m/s)	455.136
Number of rotor blades	36
Number of stator blades	46
Tip clearance(mm)	0.356

**Fig. 2:**
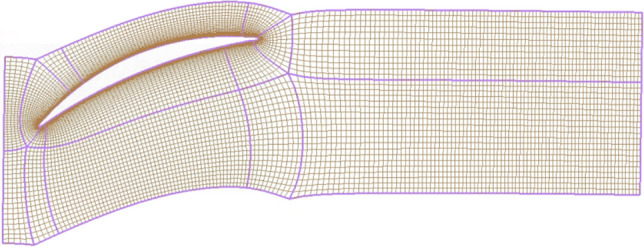
Stage37 stator blade grid.

Boundary conditions are crucial to the numerical simulation results of stage37. They affect both the results of the calculation and the convergence performance and convergence speed of the calculation process. Inlet boundary conditions specify a total temperature of 288.15K and a total pressure of 101325Pa. The boundary conditions of the rotor blades, stator blades, and hubs are set as no-slip and adiabatic. The structured grid is easier to fit into the boundary region, and it is more suitable for the computational simulation of fluids than the unstructured grid. Figure 2 shows the detailed structure grid of the stator blade channel. When the boundary conditions and grid processing are completed, the Shear Stress Transport (SST) model is employed to accurately capture adverse pressure gradient separation flow details.

The SST turbulence model ensures the stability and accuracy of $k - \omega$ model in the near-wall region while maintaining the independence of $k - \varepsilon$ from free flow in the outer boundary layer region. The governing equations of the SST turbulence model are as follows^45^

4 $$\frac{\partial (\rho k)}{{\partial t}} + \frac{{\partial \left( {\rho U_{i} k} \right)}}{{\partial x_{i} }} = \tilde{P}_{k} - \beta^{*} \rho k\omega + \frac{\partial }{{\partial x_{i} }}\left[ {\left( {\mu + \sigma_{k} \mu_{t} } \right)\frac{\partial k}{{\partial x_{i} }}} \right]$$

5 $$\frac{\partial (\rho \omega )}{{\partial t}} + \frac{{\partial \left( {\rho U_{i} \omega } \right)}}{{\partial x_{i} }} = \alpha \rho S^{2} - \beta \rho \omega^{2} + \frac{\partial }{{\partial x_{i} }}\left[ {\left( {\mu + \sigma_{\omega } \mu_{t} } \right)\frac{\partial \omega }{{\partial x_{i} }}} \right] + 2\left( {1 - F_{1} } \right)\rho \sigma_{\omega 2} \frac{1}{\omega }\frac{\partial k}{{\partial x_{i} }}\frac{\partial \omega }{{\partial x_{i} }}$$

where *F*_1_ is the first mixing function

6 $$F_{1} = \tanh \left\{ {\left\{ {\min \left[ {\max \left( {\frac{\sqrt k }{{\beta^{ * } \omega y}},\frac{500\nu }{{y^{2} \omega }}} \right),\frac{{4\rho \sigma_{\omega 2} k}}{{CD_{k\omega } y^{2} }}} \right]} \right\}^{4} } \right\}$$

in which *y* is the near-surface distance of the wall, $CD_{k\omega }$ can be expressed as follows

7 $$CD_{k\omega } = \max \Big(2\rho \sigma_{\omega 2} \frac{1}{\omega }\frac{\partial k}{{\partial x_{i} }}\frac{\partial \omega }{{\partial x_{i} }},10^{-10}\Big)$$

when *F*_1_ is set to 0, the turbulence model is essentially a $k - \varepsilon$ turbulence model. When *F*_1_ is set to 1, then the turbulence model is essentially a $k - \omega$ turbulence model. The viscosity coefficient of turbulent vortex is defined as follows

8 $$\nu_{t} = \frac{{\alpha_{1} k}}{{\max (\alpha_{1} \omega ,SF_{2} )}}$$

where S is the strain change rate tensor, $\alpha_{1} = 0.31$. *F*_2_ is the second mixing function

9 $$F_{2} = \tanh \left[ {\left[ {\max \left( {\frac{2\sqrt k }{{\beta^{ * } \omega y}},\frac{500\nu }{{y^{2} \omega }}} \right)} \right]^{2} } \right]$$

A limiter $\tilde{P}_{k}$ is used in the SST turbulence model to avoid turbulence accumulation in the stagnant region, defined as

10 $$P_{k} = \mu_{t} \frac{{\partial U_{i} }}{{\partial x_{j} }}\left( {\frac{{\partial U_{i} }}{{\partial x_{j} }} + \frac{{\partial U_{j} }}{{\partial x_{i} }}} \right) \to \tilde{P}_{k} = \min \left( {P_{k} ,10\beta^{*} \rho k\omega } \right)$$

The coefficient calculation in the SST turbulence model is as follows

11 $$\alpha = \alpha_{1} F + \alpha_{2} (1 - F)$$

where $\alpha_{1}$ represents the coefficient in the $k - \omega$ model and $\alpha_{2}$ represents the coefficient in the $k - \varepsilon$ model. The values of each coefficient in the turbulence model is defined as follows: $\beta^{*} = 0.09$, $\alpha_{1} = {5 \mathord{\left/ {\vphantom {5 9}} \right. \kern-0pt} 9}$, $\beta_{1} = {3 \mathord{\left/ {\vphantom {3 {40}}} \right. \kern-0pt} {40}}$, $\sigma_{k1} = 0.85$, $\sigma_{\omega 1} = 0.5$, $\alpha_{2} = 0.44$, $\beta_{2} = 0.0828$, $\sigma_{k2} = 1$, $\sigma_{\omega 2} = 0.856$.

**Fig. 3:**
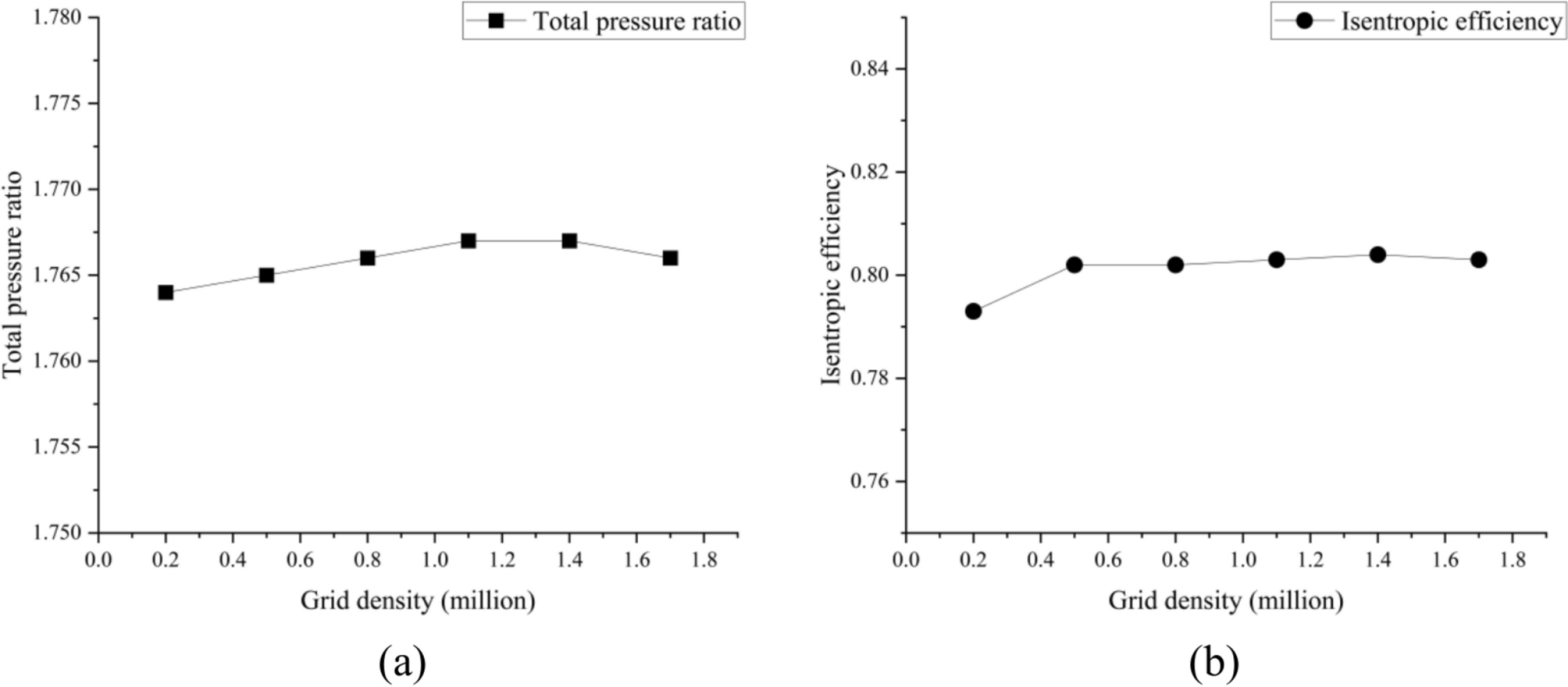
Grid-independent verification. (**a**) Total pressure ratio; (**b**) Isentropic efficiency.

Grid-independent verification is important for numerical simulations. Larger and smaller grid numbers may affect the numerical simulation of compressor. The number of grids can be adjusted by changing the number of grid nodes. Figure 3 shows the total pressure ratio and isentropic efficiency at different numbers of grids under near maximum efficiency condition. When the total number of grids is 0.8 million, the change in performance parameters becomes relatively small, indicating grid count irrelevance. This study considers the computation time and accuracy of the numerical simulation for the compressor, CFD results with the total grids number of 0.8 million can be selected as the compressor datasets source.

At the outlet boundary condition, a relatively low static pressure is prescribed to simulate the blockage boundary. Subsequently, the back pressure is gradually augmented until the calculation diverges, and the last stable computed point is identified as the stall boundary point. The static pressure value of the maximum efficiency point is located between the blockage boundary and the stall boundary.

**Fig. 4:**
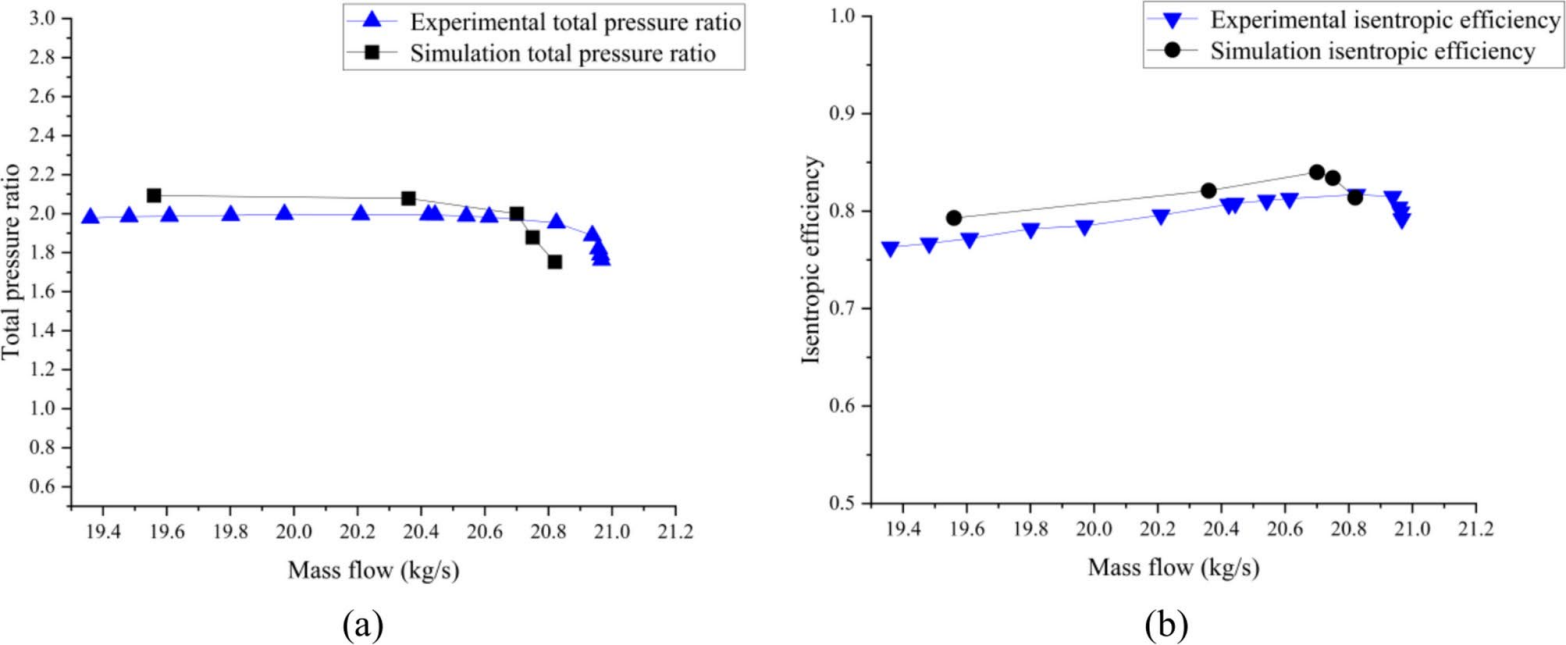
Comparison of characteristic curves between numerical simulation and experimental results: (**a**) Total pressure ratio; (**b**) Isentropic efficiency.

Consequently, numerical simulation results of Mach number are obtained for near blockage condition, near maximum efficiency condition and near stall condition. To validate the accuracy of the numerical calculations, total pressure ratio curve and isentropic efficiency curve are compared with experimental data^44^ published by NASA at Fig. 4. It is observed that the total stage pressure ratio and isentropic efficiency obtained from numerical calculations exhibit some deviations from the experimental data. However, the overall trend of the simulated characteristic curve aligns closely with the experimental results.

### Dataset composition

**Fig. 5:**
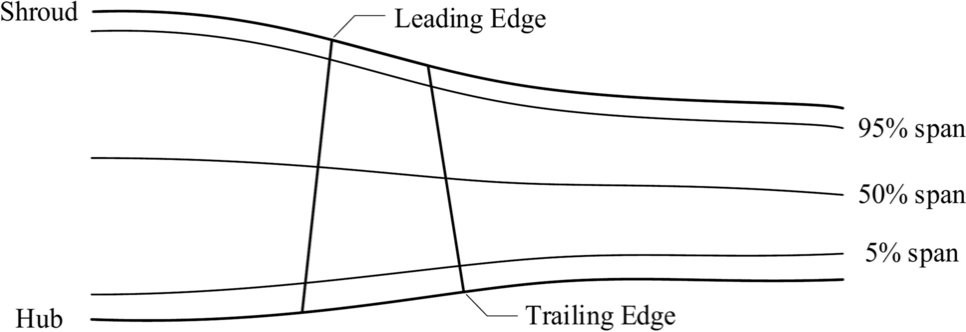
Diagram of different span heights of blades.

As depicted in Fig. 5 blade distortion leads to variations in the flow field across different spans. The compressor blade data consist of Mach number field for different span and blade geometry images.

In image processing, training data is typically formatted as input for CNNs in the form of images. Prior to inputting training data, image preprocessing is commonly performed to standardize the distribution of training data, thereby effectively mitigating the risk of gradient vanishing. The preparation of input images is pivotal in handling compressor blade data. A grayscale image contains only brightness information, where each pixel value denotes the grayscale level at the corresponding position in the image. Grayscale levels are typically represented as integers ranging from 0 to 255, with 0 representing black and 255 representing white. Grayscale images encapsulate fundamental properties of the object in the image, such as shape, texture, and edges. The input of a grayscale image is a two-dimensional matrix, while the input of an RGB image consists of three two-dimensional matrices. Grayscale images can reduce computational complexity. For instance, a grayscale image has 256 dimensions, an RGB image has over 16 million dimensions. With colored images, the number of features and computational load increase exponentially. The blade data in this work is represented as grayscale images, which can be directly utilized as input for the CNN model. Grayscale processing enhances training efficiency by reducing computational overhead.

To ensure the robustness of prediction model, the Mach number field is normalized. By normalizing the Mach number data, subsequent network processing is expedited. Following normalization, the dataset is divided into a train dataset and a test dataset. Approximately 80% of the data is earmarked for the train dataset so that prediction model can be built. The remaining portion constitutes the test dataset, which serves for unbiased evaluation of the model’s quality during training, facilitating the detection of overfitting.

### Neural network architecture

**Fig. 6:**
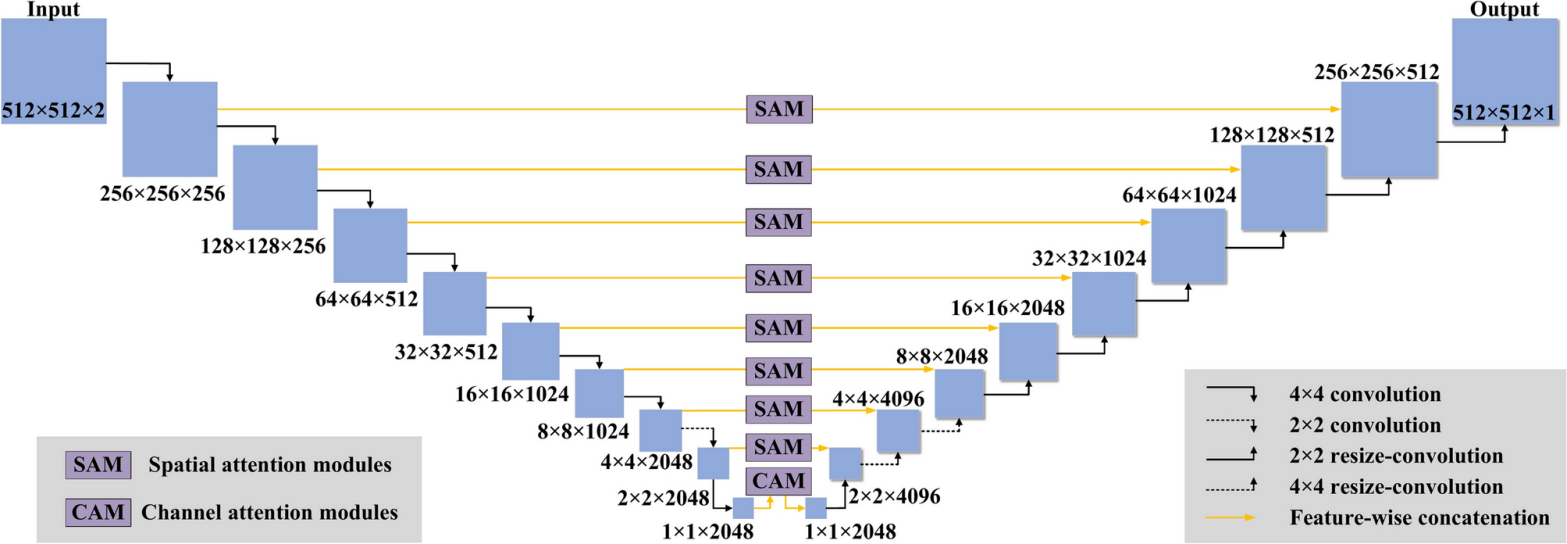
Architecture of neural network model.

As depicted in Fig. 6 the neural network model is improved on the basis of U-Net architecture^46^. The network exhibits a typical U-shaped structure, facilitating the transformation of spatial information into extracted features through convolutional layers. Feature-wise concatenation from input to output feature channels is introduced, ensuring that feature information of varying sizes is accessible in the output layer to bolster prediction accuracy. Different from the standard U-Net architecture, attention modules (AMs) are combined to squeeze the spatial dimension of the input feature map. Specifically, a channel attention module (CAM) is introduced at the bottom to improve representation power of network, and eight spatial attention modules (SAMs) are introduced in all other Feature-wise connections to improve the network’s ability to learn data distribution. These attention modules play a crucial role in enhancing the integration of fine-grained and coarse-grained information within the Feature-wise concatenation, thereby improving the model’s effectiveness in capturing intricate details and global information.

Neural network, a fundamental component of DL, serve as a general method for regression towards arbitrary nonlinear function *f*. Deep feedforward networks, also referred to as multi-layer perceptrons (MLPs), surpass the constraints of linear models by incorporating one or more hidden layers. MLPs are considered prototypes for DL models^47^. MLPs are good at capturing intricate interactions among inputs through hidden neurons, which are capable of performing arbitrary calculations based on input values. This flexibility allows for the design of hidden nodes to approximate complex function *f* efficiently.

For example, $y = f(x)$ maps input *x* to prediction *y*. A feedforward network defines a mapping $y = f^{ * } (x;\theta )$. It is optimized to find the parameter $\theta$ that yield the best approximation of the function *f*. In the feedforward network, information flow initiates from *x* , traverses through the evaluated function, undergoes intermediate calculations, and ultimately arrives at the output *y*, as described by Goodfellow et al.^48^.

Neural networks are often depicted as combinations of several functions arranged in a directed acyclic graph, which can be conceived of $f^{ * } (x)$ as comprising the connection of *n* different functions in a chain, so that

12 $$f^{ * } ({\mathbf{x}}) = f^{(n)} ( \cdots (f^{(2)} (f^{(1)} ({\mathbf{x}}))))$$

where $f^{(1)}$, $f^{(2)}$, … and $f^{(n)}$ are different functions corresponding to each layer of the network. The depth of the model corresponds to the total length of the above chains. The first and last layers serve as the input and output layers, respectively. With any intermediate layers are termed hidden layers.

Neural networks use interconnected neurons to model the objective function. In the subsequent discussion, neural networks are represented by *f*. Neurons typically aggregate values from previous neurons and apply activation functions *g*. Activation functions determine whether a neuron should be activated by calculating a weighted sum and adding a bias, *g* convert the input signal into a differentiable operation of the output, and most activation functions are nonlinear. These activation functions are crucial to introduce non-linearity, and effectively allow neural networks to approximate arbitrary functions. Typical choices for *g* include hyperbolic tangent, sigmoid and ReLU functions.

Compared with other activation functions, ReLU has distinct advantages. It circumvents the issue of gradient vanishing. The computation of ReLU is straightforward, devoid of complex exponential calculations, resulting in rapid convergence and excellent performance across various forecasting tasks. As shown in Eq. (13), when the input is negative, the derivative of ReLU function is 0, and when the input is positive, the derivative of ReLU function is 1.

13 $${\text{Re}} LU(x) = \left\{ {\begin{array}{*{20}c} x & {x > 0} \\ 0 & {x < 0} \\ \end{array} } \right.$$

The previous function $f^{ * } (x)$ fitting procedure can be described as: for a layer *l* in the network, the output of the i’th node $a_{i,l}$ is computed with

14 $$a_{i,l} = g\left( {\sum\limits_{j = 0}^{{n_{l - 1} }} {w_{ij,l - 1} a_{j,l - 1} } } \right)$$

in which, $n_{l}$ indicates the number of nodes in each layer. To incorporate the bias, it is assumed that $a_{0,l} = 1$ for all *l*. This bias is essential for enabling nodes to transfer input to the activation function. This common formula is used to represent all the degrees of freedom in terms of the weight vector **w**. Therefore, conventional weights and biases are not clearly distinguished below. Equation (14) can be rewritten with the weight matrix **W** as

15 $${\mathbf{a}}_{l} = {\mathbf{g}}({\mathbf{W}}_{l - 1} {\mathbf{a}}_{l - 1} )$$

where **g** is a component to the input vector. Note that without the nonlinear activation function **g**, a single matrix **W**_0_ can be used to represent the entire network

16 $${\mathbf{a}} = {\mathbf{W}}_{0} x$$

During forward propagation, the results of each layer in the neural network are computed and stored sequentially, from the input layer to the output layer. To facilitate weight calculation, we need to specify a loss function $L({\mathbf{y}},f(x,w))$ for the learning process. This loss function is tailored to the specific problem and typically has two purposes. One is to evaluate the quality of the output generated relative to **y**, and the other is to minimize potential overfitting through regularization. According to the definition of *L*_2_ regularization, given the hyper- parameter λ, the regularization term is

17 $$s = \frac{\lambda }{2}\sum\limits_{l} {w_{l}^{2} }$$

the regularization loss of the model with a given data sample is

18 $$J = L + s$$

where *J* is the regularization loss function. The loss function *L* requires first-order differentiability so that its gradient $\nabla_{y} L$ propagates back into the network to compute the weight gradient $\nabla_{w} L$.

Back propagation refers to the method of computing the gradient of neural network parameters. Essentially, the method follows the chain rule of calculus, traversing the network from the output layer to the input layer in reverse order. During this process, the algorithm retains intermediate variables (partial derivatives) of the calculation. The partial derivative of the regularization loss function *J* can be expressed as

19 $$\frac{\partial J}{{\partial w_{l} }} = \frac{\partial L}{{\partial w_{l} }} + \lambda w_{l}$$

During the training of neural networks, after initializing the model parameters, we iteratively perform forward propagation and back propagation, updating the model parameters using the gradients obtained from back propagation. Note that back propagation reuses the intermediate values stored during forward propagation to avoid redundancy. Consequently, these intermediate values must be retained until the back propagation process is completed. This is one of the reasons that training requires more video memory than prediction. In addition, the size of these intermediate values is roughly proportional to the number of network layers and the batch size. Therefore, using larger batches to train deeper networks may increase the risk of experiencing out of memory error.

**Fig. 7:**
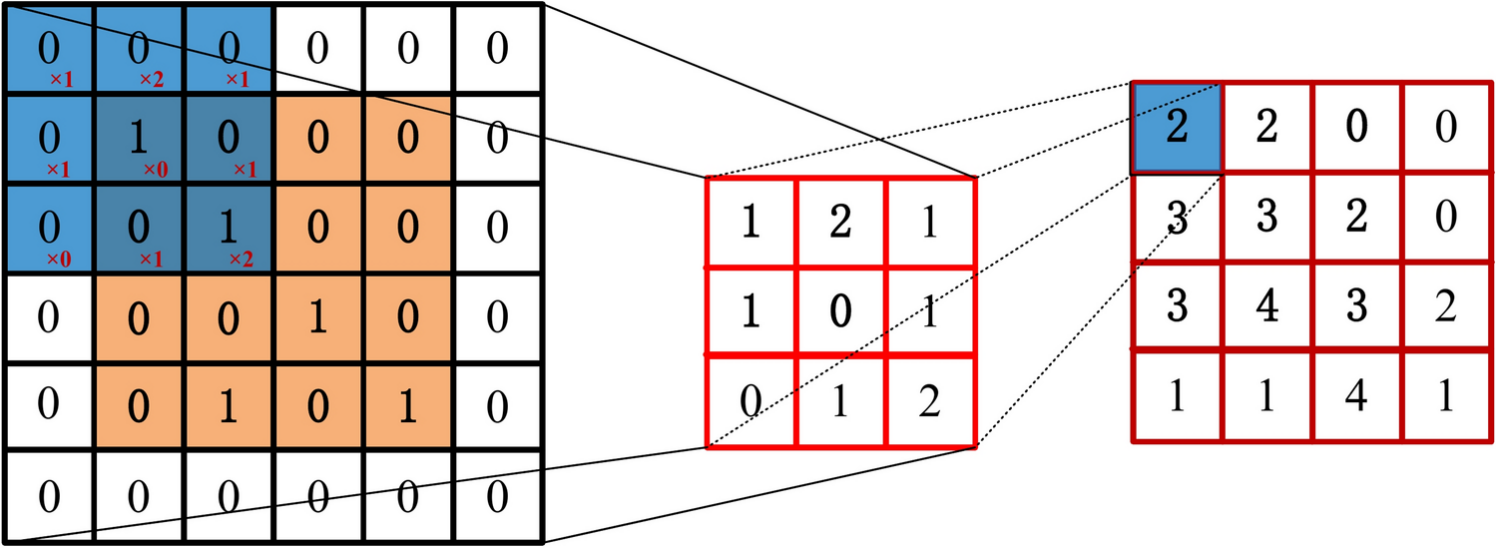
An example of convolution operation.

The convolutional layer serves as a central component in many deep learning algorithms, offering flexibility in adjusting the dimensionality of data through setting padding and stride parameters. For a given matrix **A**, the general expression of convolution to **A** is^49^

20 $${\mathbf{B}} = {\mathbf{wA}}$$

where **B** is the convoluted matrix and **w** is the convolution kernel. As illustrated in Fig. 7, input is a 4 × 4 matrix (orange area), with a padding of 1 and a stride of 1. The bias is set to 0, and the convolution kernel is 3 × 3 matrix (blue area). In the convolution layer, the input is cross-correlated with the convolution kernel weights. The kernel slides over the input domain with a stride of 1 to perform convolution, and the output is obtained by adding the scalar bias. Therefore, the two parameters trained in the convolution layer are the convolution kernel weights and the scalar bias. In our case, the padding value* P* can be computed by

21 $$P = \frac{K}{2}$$

where *K* is the convolution kernel size and *P* is always an integer.

In image processing tasks, it is necessary to gradually reduce the spatial resolution of hidden layer representations and aggregate information. This process ensures that when the neural network is trained, each neuron becomes more sensitive to the characteristic parameters of the input information. To achieve this, neural networks commonly employ pooling layers to aggregate information. Similar to convolutional layers, pooling layers utilize a fixed-shaped window that slides over all areas of the input based on its stride size. At each position traversed by the fixed-shaped window, the pooling layer computes an output. However, unlike the cross-correlation calculations between the input and the convolution kernel in the convolutional layer, pooling layers do not contain any parameters. Instead, pooling operations are deterministic, and the maximum or average value of all elements in the receptive field is typically calculated. These operations are known as maximum pooling layer and average pooling layer, respectively. They are used to downsample the input data, reducing its spatial dimensions while retaining important features. This downsampling process helps control overfitting, reduces computational complexity, and improves the efficiency of subsequent layers in the neural network.

**Fig. 8:**
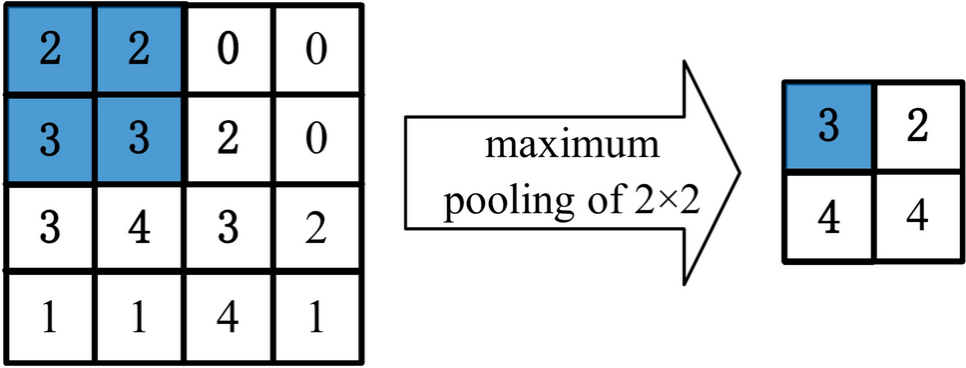
An example of maximum pooling operation.

In both cases, at each location reached by the fixed-shaped window to calculate the maximum or average value of the tensor entered. Figure 8 illustrates the process of maximum pooling with a 2 × 2 window. The specific calculation of the maximum or average value depends on whether the maximum pooling layer or average pooling layer is utilized.

**Fig. 9:**
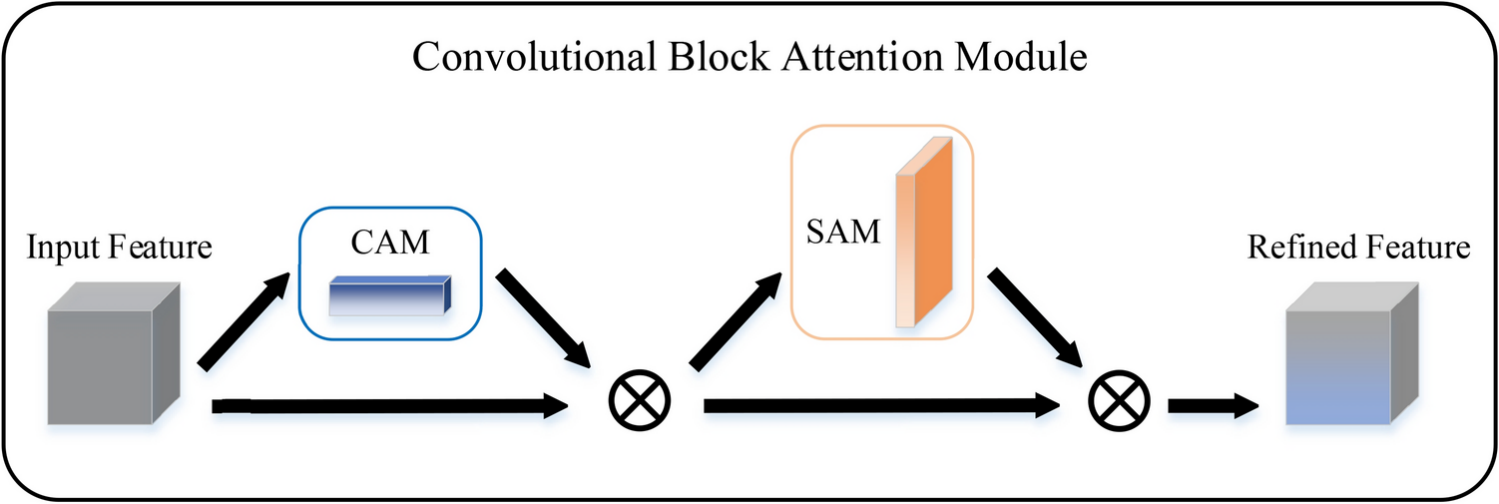
Convolutional block attention module.

In a departure from previous approaches in deep learning-based CFD approximation, attention mechanisms are incorporated into learning framework. When some areas of fluid flow tend to contain more complex and important information than others, attention mechanisms can be used to enhance the feature extraction effect of the network, especially when the flow exhibits rapid changes. Hu et al.^50^ proposed the attention mechanism guides the network to focus on the separation region and the supersonic flow region. As illustrated in Fig. 9, two lightweight attention mechanisms are integrated into the prediction model, namely the Channel Attention Module (CAM) and the Spatial Attention Module (SAM), which are extensions of the work by Woo et al.^51^.

**Fig. 10:**
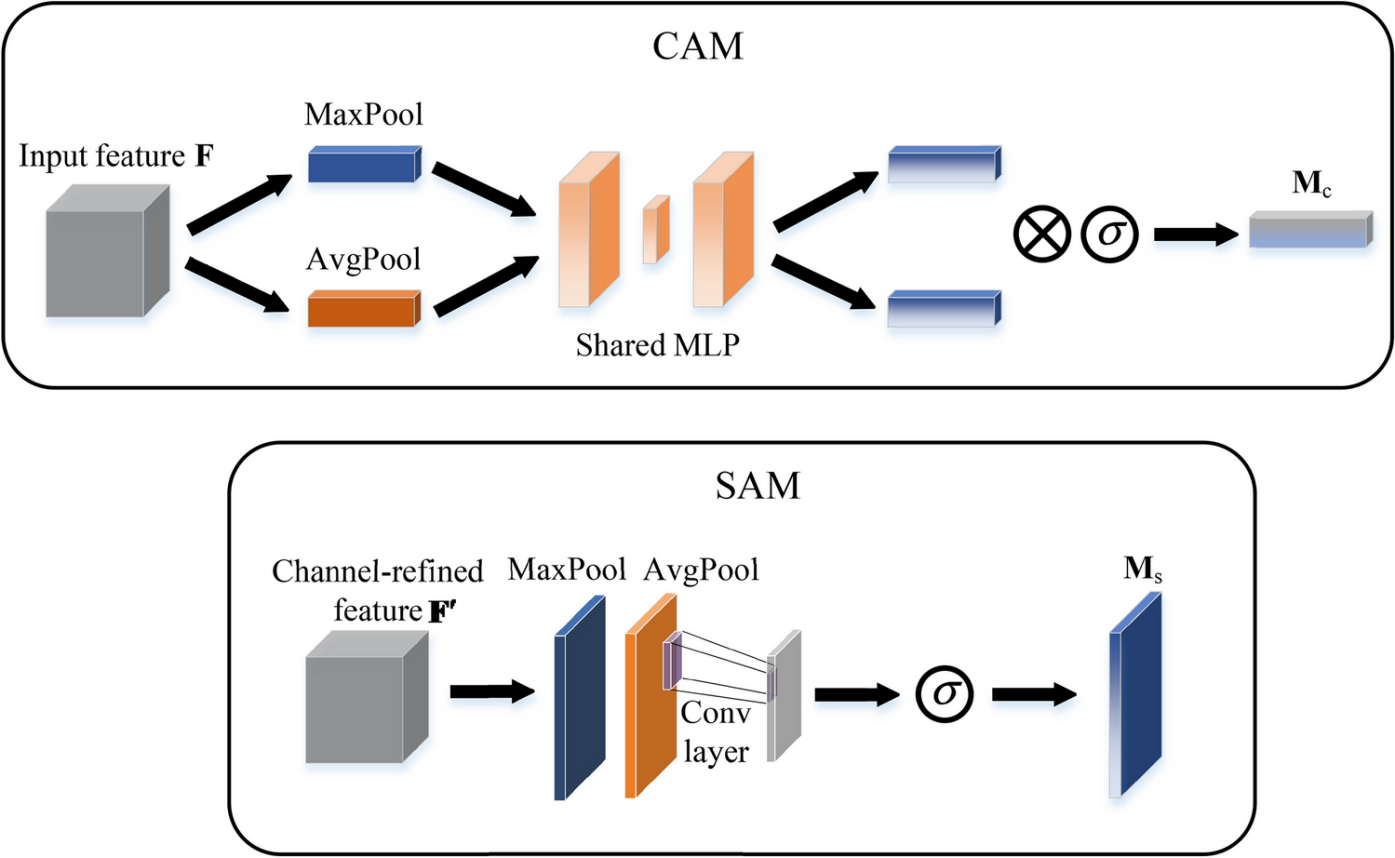
Diagram of each attention sub-module.

The CAM and the SAM are capable of extracting discriminative features from the channel and spatial domains. Figure 10 depicts the computation process of each attention map. The subsequent equations illustrate the operational principles of these two attention modules

22 $$F_{{\text{c}}} = M_{{\text{c}}} \left( {\mathbb{F}} \right) \otimes {\mathbb{F}},$$

23 $$F_{{\text{s}}} = M_{{\text{s}}} \left( {\mathbb{F}} \right) \otimes {\mathbb{F}},$$

24 $$M_{{\text{c}}} \left( {\mathbb{F}} \right) = \sigma \left( {{\text{MLP}}\left( {{\text{GAP}}\left( {\mathbb{F}} \right)} \right) + {\text{MLP}}\left( {{\text{GMP}}\left( {\mathbb{F}} \right)} \right)} \right),$$

25 $$M_{{\text{s}}} \left( {\mathbb{F}} \right) = \sigma \left( {{\text{Conv}}\left( {{\text{GAP}}_{{\text{c}}} \left( {\mathbb{F}} \right) \oplus {\text{GMP}}_{{\text{c}}} \left( {\mathbb{F}} \right)} \right)} \right)$$

where $\otimes$ and $\oplus$ represent element-wise multiplication and channel-wise concatenation, respectively. Here, ${\mathbb{F}} \in {\mathbb{R}}^{C \times H \times W}$ denotes the input feature map, while $M_{{\text{c}}} \in {\mathbb{R}}^{{C \times {1} \times {1}}}$ and $M_{{\text{s}}} \in {\mathbb{R}}^{{{1} \times H \times W}}$ represent the CAM and the SAM, respectively. The intermediate results $M_{{\text{c}}} \left( {\mathbb{F}} \right)$ and $M_{{\text{s}}} \left( {\mathbb{F}} \right)$ need $\otimes$ with ${\mathbb{F}}$ itself to match the dimension of the original input, yielding the outputs *F*_c_ and *F*_s_. Equations (24) and (25) delineate the specific operations within CAM and SAM.

CAM initially conducts global average pooling (GAP) and global max pooling (GMP) along the spatial axis of the input feature map to generate a channel vector. Subsequently, this vector is fed into an MLP comprising one hidden layer to estimate attention across channels. SAM similarly incorporates global pooling operations, however, SAMs are conducted along the channel axis, denoted as ${\text{GAP}}_{{\text{c}}}$ and ${\text{GMP}}_{{\text{c}}}$. The outcomes from ${\text{GAP}}_{{\text{c}}}$ and ${\text{GMP}}_{{\text{c}}}$ are concatenated and then subjected to a convolution operation to produce a spatial attention map with one channel. Both CAM and SAM are subsequently subjected to the sigmoid function $\sigma$ for normalization, and the relatively small overhead of attention mechanism (AM) in terms of parameters and computation determines the application of prediction model.

### Supervised training

After conducting experiments with various architectures involving different numbers of Feature-wise concatenations, the U-Net architecture yielded high-quality results with relatively low memory requirements. Therefore, this article will focus on the most successful model. Table 2 presents the specific parameters of several cases, with a learning rate of 6 × 10^−4^ across all cases. Case 1 emerged as the optimal model, and thus, case 1 is selected as the final network structure. This U-Net architecture is a specialized instance of an encoder-decoder architecture. In the encoding phase, cross-line convolution is employed to progressively reduce the image size by a factor of 2. The number of feature channels in each convolutional layer is determined by the Channel-Exponent (base 2). For example, there are 16 channels when Channel-Exponent is 4. The feature channel count is doubled after every two convolution layers. This design facilitates extraction of increasingly large-scale and abstract information across a growing number of feature channels.

**Table 2:** Specific parameters of several cases.

	Number of layers	Iterations	Channel-Exponent	Resolution	Mean absolute error (%)
Case1	19	40,000	8	512 × 512	0.72898
Case2	19	20,000	8	512 × 512	1.98572
Case3	15	40,000	6	512 × 512	1.59285

In the decoding phase of network, the opposite operation is performed, where spatial resolution is increased, and the number of feature layers is reduced using average pooling layers. Feature-wise concatenations connect all channels of the encoded branch to corresponding branch of the decoded part, effectively doubling the number of channels per decoded block. These Feature-wise concatenations enable the network to consider low-level input information when reconstructing flow field information at the decoding layer. In addition to the nonlinear activation function, each part of the network includes a convolution layer and a batch normalization layer. For a standard U-Net with a weight of 7.7m, a 512 × 512 × 2 input is transformed into a single data point with 2048 features using nine convolution blocks, typically employing convolution cores of size 4 × 4 (only inner three layers of the encoder use 2 × 2 convolution cores). The leaky-ReLU function with a slope of 0.2 is utilized as activation function at encoding layer, while ReLU activation function is applied at the decoding layer. The decoder portion decodes objective function using nine additional symmetric layers, with an expected dimension of 512 × 512 × 1.

This work utilizes PyTorch deep learning framework for model training. The prediction model is trained using Adam optimizer, and learning setting follows supervised learning principles, using a simple L_1_ loss

26 $$L = \frac{1}{n}\sum\limits_{i = 1}^{n} {\frac{{\left| {\tilde{y}_{i} - y_{i} } \right|}}{{y_{i} }}}$$

where $\tilde{y}_{i}$ is output of the prediction model.

**Fig. 11:**
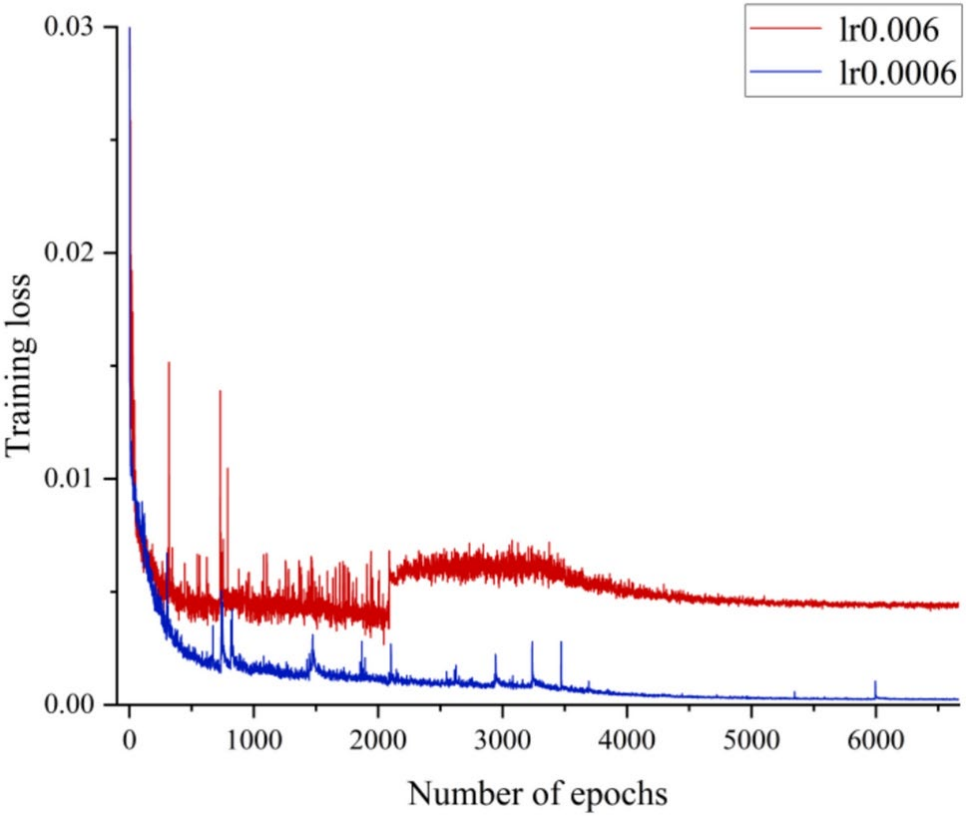
Training loss curve of case 1 (near the maximum efficiency condition).

To establish the training methodology, several fundamental training parameters are assessed initially, including learning rate, learning rate decay, and input data normalization. Among these parameters, the learning rate of optimizer stands out as one of the most critical aspects of deep learning. Learning rate determines the step size at which optimizer updates the weights of neural network based on gradient calculated from a small batch of data. Typically, the functions fitted by deep neural networks are nonlinear with large minima. Consequently, choosing an appropriate learning rate is essential. A small learning rate may result in slow convergence or being trapped in local optima, while a large learning rate can impede convergence altogether. Figure 11 illustrates the training loss curve of case1 under the condition of 50% span near maximum efficiency. It is evident that the training loss increases significantly when the learning rate is increased by an order of magnitude. Thus, selecting the appropriate learning rate is crucial, depending on various factors such as training objectives and network structures.

**Fig. 12:**
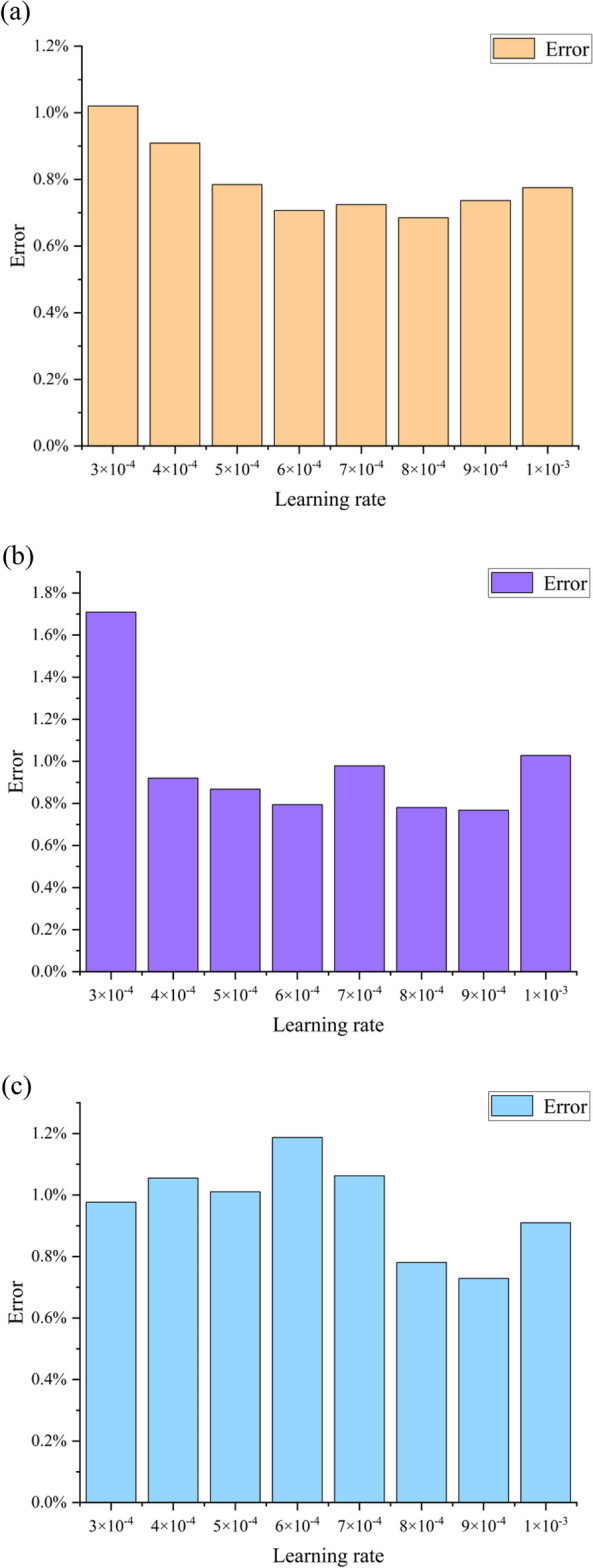
The average absolute error at different learning rates (**a**) Near blockage condition; (**b**) Near maximum efficiency condition; (**c**) Near stall condition.

The learning rates selected in the range of 3 × 10^−4^ to 1 × 10^−3^, all cases have good convergence. Figure 12 shows the influence of the learning rate on the prediction accuracy under three different working conditions.

Given the complexity of typical neural network learning tasks, it’s crucial to identify the best parameters for training. An extensive hyper-parameter search for learning rates has been conducted to evaluate their impact on prediction accuracy. Specifically, a learning rate of 0.0008 is utilized in the near blockage condition, 0.0009 in the near maximum efficiency condition, and 0.0009 in the near stall condition, with a batch size of 10. Furthermore, using learning rate decay to reduce the learning rate during training helps to stabilize the results and reduce the performance variance. When other parameters were well-selected, the effect of learning rate decay wasn’t substantial. However, the learning rate is reduced to 10% of its initial value in the second half of the training iterations to further enhance stability.

## Results and discussions

**Fig. 13:**
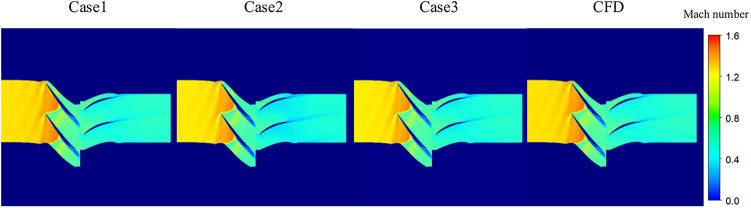
Prediction results of 50% span in three cases under near maximum efficiency.

The model optimization process has been detailed previously. Next, qualitative and quantitative analysis of the results is conducted, and the accuracy and performance of the model are discussed. Figure 13 depicts the prediction results of 50% span under near maximum efficiency condition for the three selected cases. In alignment with the average absolute error of the three cases presented earlier, Case 1 emerges as the optimal model.

**Fig. 14:**
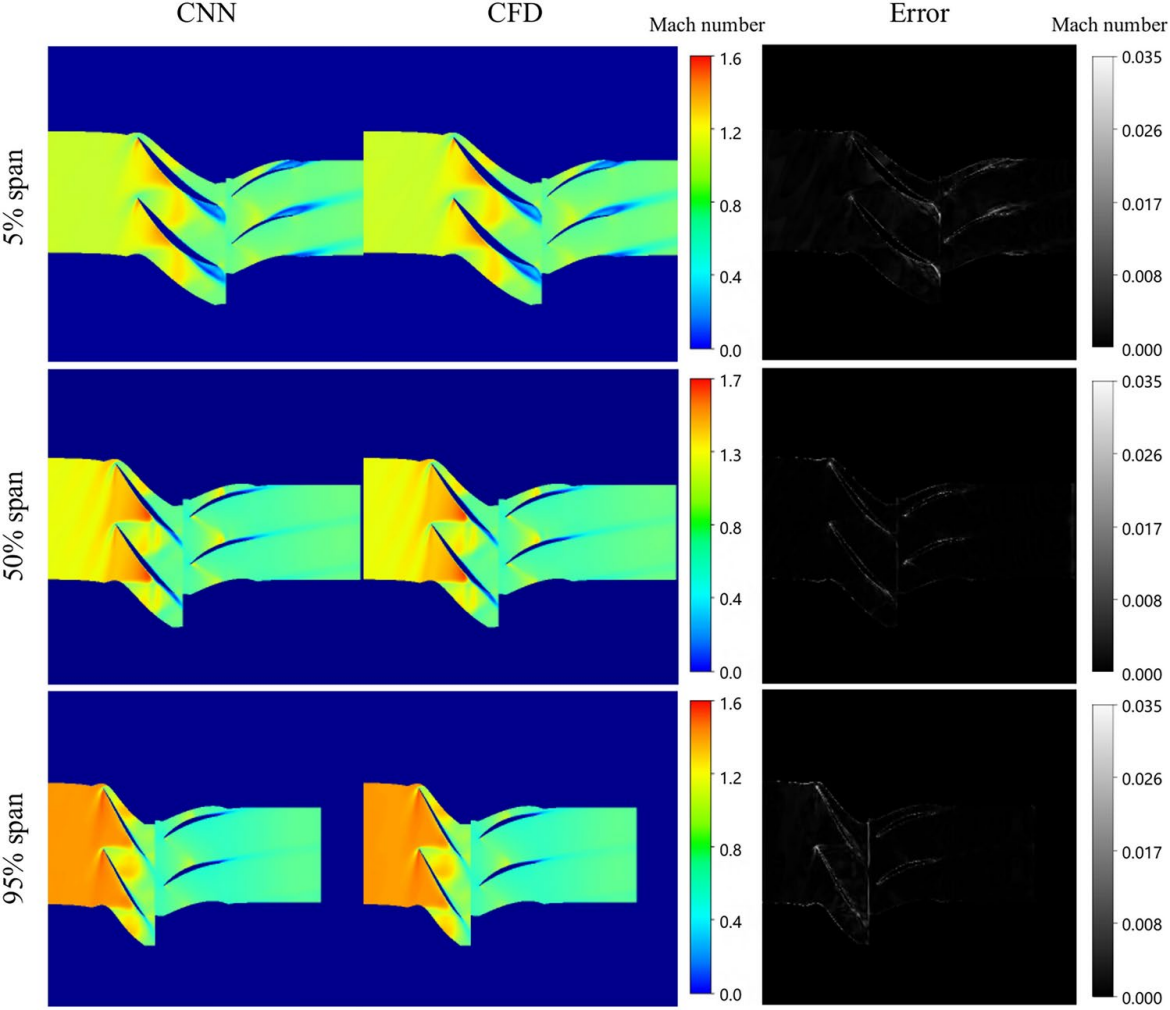
Comparison between ground-truth CFD and model prediction(Near blockage condition).

By comparing the results of numerical simulation with those of prediction, the performance of prediction model is evaluated. The capability of the proposed model in providing efficient approximations of steady-state flow solutions is demonstrated. Without additional explanation, the following results are all the prediction results of case 1 model. Figure 14 displays the Mach number flow field prediction results at various span under near blockage condition, contrasted with the ground-truth CFD solution. The left column showcases the predicted results for the Mach number. The middle column presents the ground-truth CFD solution. Absolute errors are listed in the right column for comparison.

In the near blockage condition, a substantial air separation region is observed at the trailing edges of both the rotor and stator blades at 5% span. This phenomenon arises from the reduced back pressure in the blockage condition and the resultant accelerated airflow within the cascade channel. Upon reaching the speed of sound, shock waves form within the cascade channel, interacting with the boundary layers of the blade suction surfaces, thereby leading to boundary layer separation at the suction surfaces of both the rotor and stator blades. Consequently, a low-speed blockage zone is formed.

Slight boundary layer separation is evident at the upper section of the 50% span, localized at the trailing edges of the suction surfaces of both the rotor and stator blades, albeit to a lesser extent compared to the root of the blades. Throughout the rotor blade, gas flow is rapid, with a considerable area exhibiting supersonic velocities.

At the 95% span, the supersonic flow region predominates throughout the rotor blade channel. As the span height of the rotor blade channel increases, notably compared to the 5% and 50% span, the circumferential velocity of the blade rotation escalates. Consequently, the gas flow within the compressor’s supersonic region intensifies correspondingly.

**Fig. 15:**
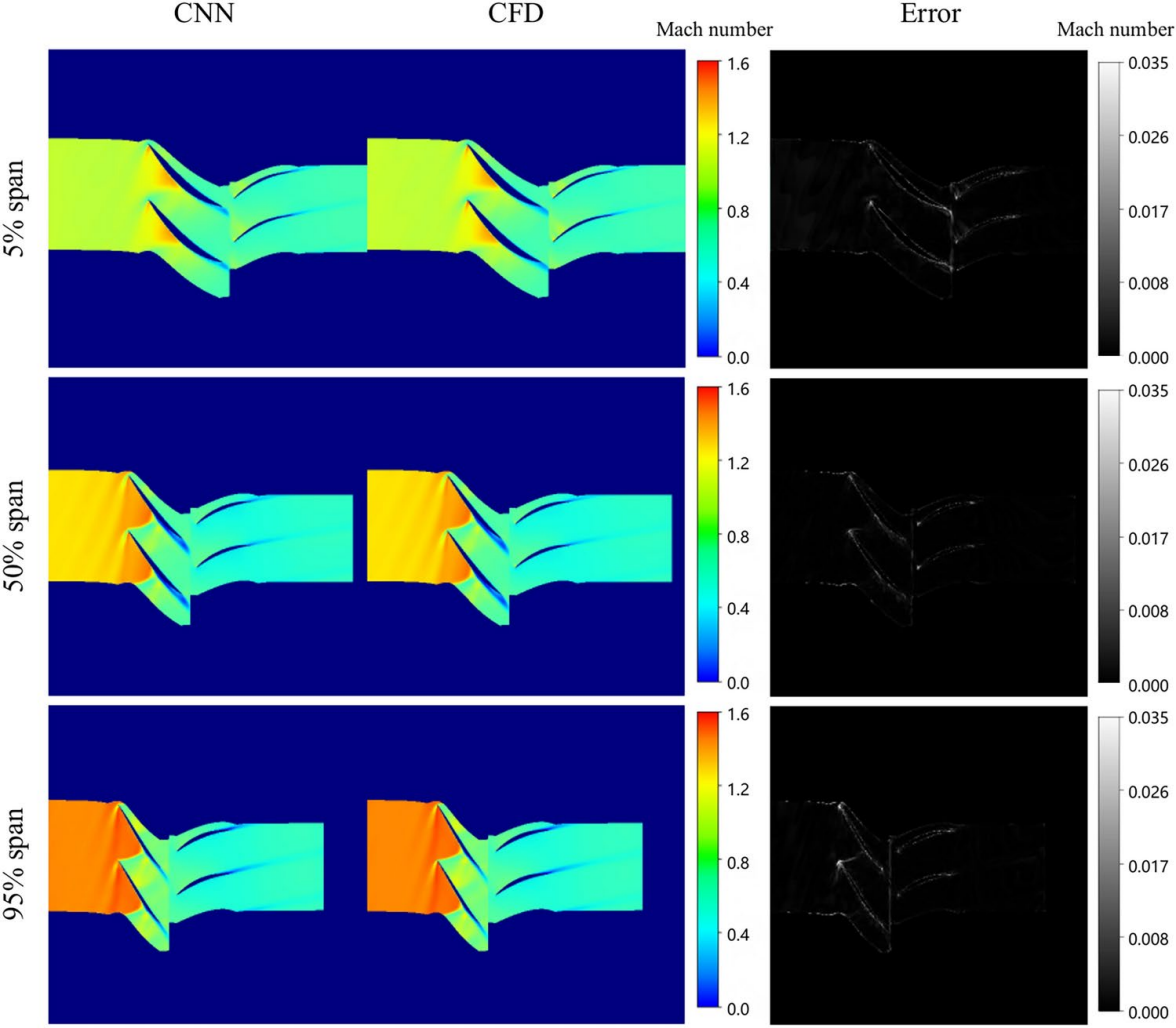
Comparison between ground-truth CFD and model prediction (Near maximum efficiency condition).

The predicted results under the condition of near maximum efficiency are shown in Fig. 15. At the 5% span, the flow within the entire blade root region of the cascade channel appears smooth, with only a small region of boundary layer separation observed at the tail of both the rotor and stator blades. However, compared to the near blockage condition, the low-speed region is notably reduced due to the absence of shock waves.

At the 50% span, a shock wave is observed in approximately 50% of the rotor blade channel’s chord length, with its position relatively shifted compared to the near blockage condition. Under the influence of the shock waves, boundary layer separation occurs on the suction surface of the rotor blade after 50% chord length. Meanwhile, the flow within the stator blade channel remains relatively stable, although with a minor wake is observed at the tail of the blade.

At the 95% span, the airflow within the rotor blade channel exhibits relative stability. Following shock wave diffusion, the supersonic airflow within the rotor blade decelerates, transitioning to subsonic velocity. This subsonic flow then enters the stator blade channel, where further deceleration and diffusion occur within the diffusion channel of stator blade.

**Fig. 16:**
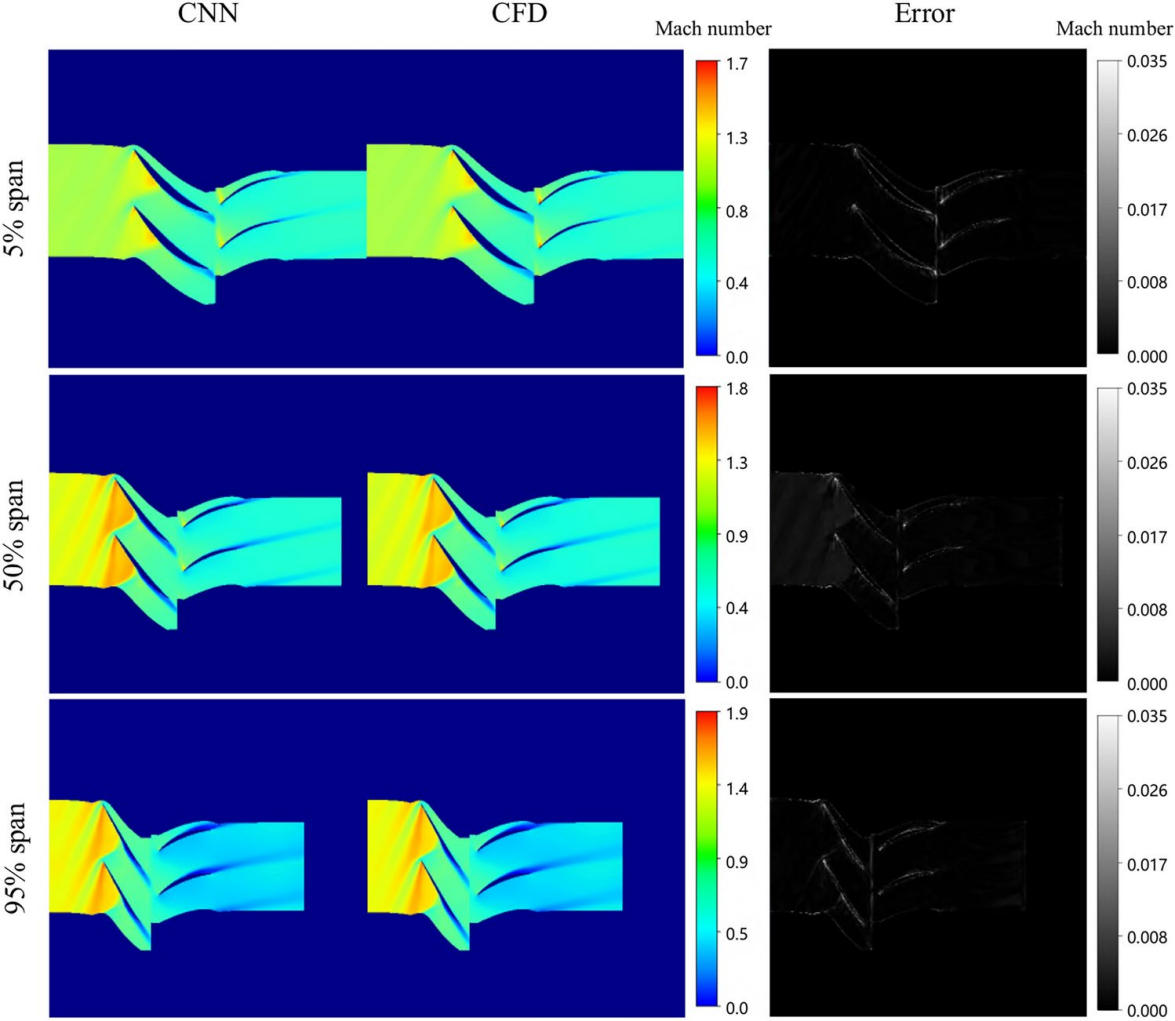
Comparison between ground-truth CFD and model prediction (Near stall condition).

The predicted results under the condition of near stall are shown in Fig. 16. At the 5% span, the increased back pressure leads to a rapid drop in axial airflow velocity. Consequently, the inlet angle of attack for the stator blade increases, contributing to a substantial amount of boundary layer flow at the root of the stator blade. This exacerbates flow conditions at the root, ultimately resulting in significant flow separation at the root of the stator blade.

At the 50% span, the shock wave position on rotor blade is situated at approximately 40% of the chord length on the suction surface side. Compared with the near blockage condition and the near maximum efficiency condition, the shock wave position of rotor blade is the most forward, primarily due to a significant adverse pressure gradient within the compressor channel under near stall conditions, leading to extensive flow separation at the tail of stator blade.

At the 95% span, as compressor load increases, the intensity of shock waves within the channel also escalates. Eventually, the shock wave detaches from the leading edge of the blade. Subsequent strong interaction between the blade tip leakage vortex and the channel shock wave distorts the shock wave shape, resulting in a large low-speed area behind the shock wave near the pressure surface of adjacent blades. This phenomenon is attributed to the disruption of the blade tip leakage vortex following interference from the shock wave. These low-energy fluids constrict the flow channel in the blade tip area, impeding gas flow and contributing to blockage in the blade tip area.

**Fig. 17:**
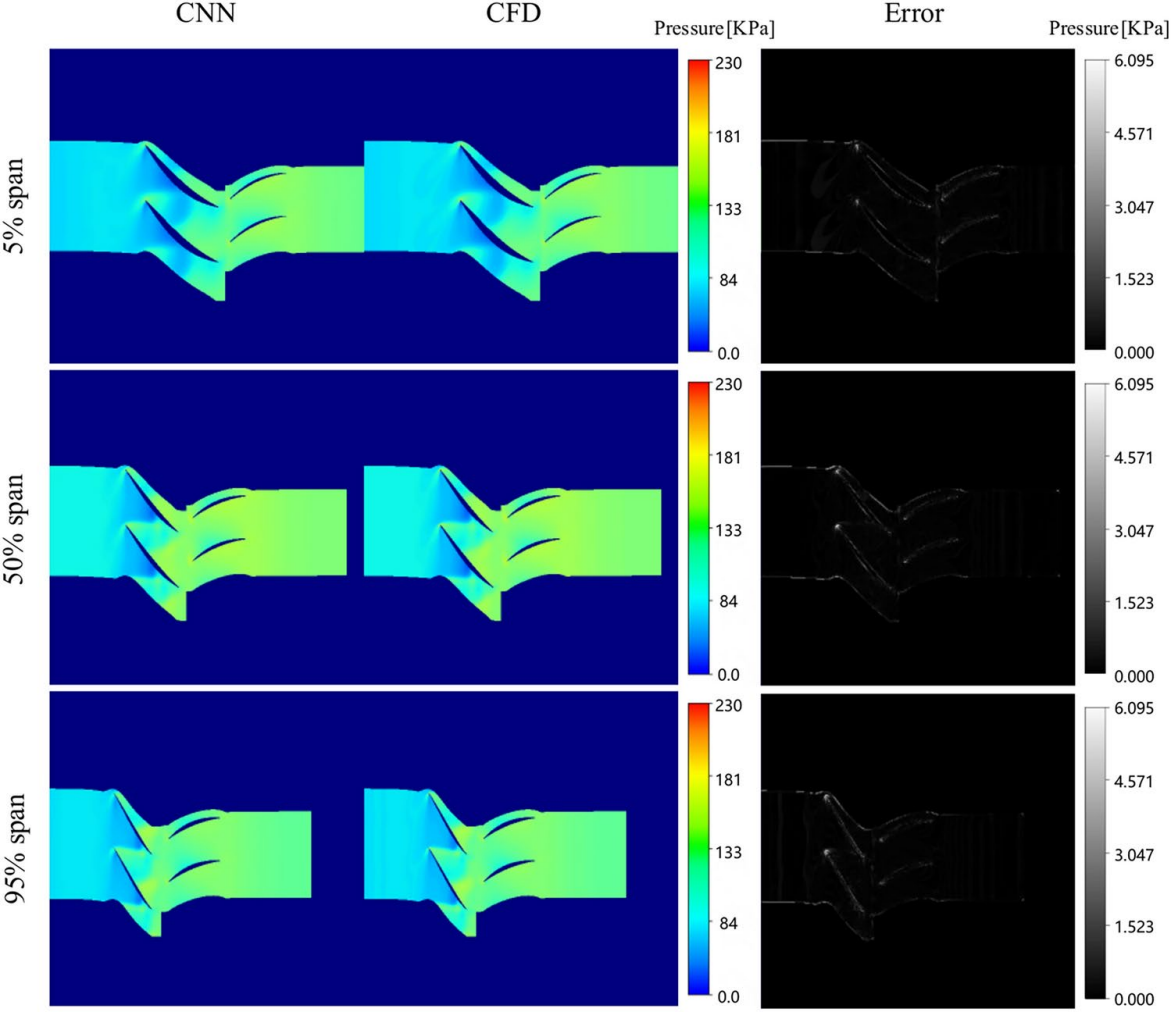
The prediction results of blade pressure field under near maximum condition.

The prediction results of blade pressure field under near maximum condition are shown in Fig. 17. The average absolute error is 0.599% and the maximum error of a single sample is 2.265%. It can be seen that the prediction model has good generalization ability to predict other variables.

Table 3 presents the mean absolute error of the optimal model across all results, as well as the maximum absolute error observed for a single sample. This quantitative analysis provides an evaluation of the performance of the prediction model under various spans.

**Table 3:** Errors of three different conditions.

	Near blockage condition		Near maximum efficiency condition		Near stall condition	
Type	Mean	Maximum	Mean	Maximum	Mean	Maximum
Value	0.685%	2.188%	0.768%	1.975%	0.729%	2.443%

The mean absolute error under three different working conditions is less than 1%, and the maximum error of any sample under each working condition is less than 3%. This level of accuracy enables thorough analysis of the aerodynamic performance of compressor blades, meeting the stringent demands of engineering applications. It is worth noting that the CNN solutions closely reflect the data distribution of the ground-truth CFD data. The ability of the prediction model to capture separation areas and supersonic flow areas under various conditions demonstrates its effectiveness in approximating complex flow phenomena. Moreover, the minimal discrepancies in mean and standard deviation between the CNN solution and CFD simulation underscore the reliability and accuracy of the prediction model. Overall, these findings validate the suitability of the proposed CNN-based approach for approximating flow fields in computational fluid dynamics applications.

On the basis of the original solid wall casing, UG was used to model the circumferential groove casing treatment. This chapter designs 5 kinds of circumferential grooves in different positions by adding circumferential grooves in different positions. The specific design parameters of the 5 kinds of circumferential grooves are shown in Table 4.

**Table 4:** The design scheme of the circumferential groove casing treatment.

Casing treatment scheme	Position (%)	Groove width (mm)	Groove depth (mm)
C1	50	3	3.56
C2	40		
C3	30		
C4	20		
C5	10		

**Fig. 18:**
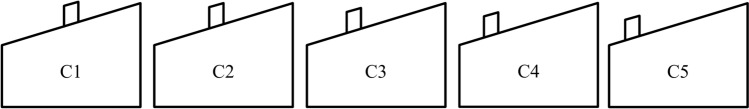
Schematic diagram of the calculation scheme.

The processing starting positions of the designed circumferential groove casing treatments are located at 50%, 40%, 30%, 20% and 10% of the relative chord length of the tip of the blade, respectively. These circumferential grooves with different axial positions have the same groove width and groove depth, where the groove width is 10.84% of the axial chord length of the blade tip, and the groove depth is 10 times of the tip clearance value. Figure 18 shows the two-dimensional diagram of circumferential groove casing processing. For the convenience of analysis, the working condition of the solid wall casing was abbreviated as SW, and the other five working conditions were abbreviated as C1, C2, C3, C4 and C5 respectively.

**Fig. 19:**
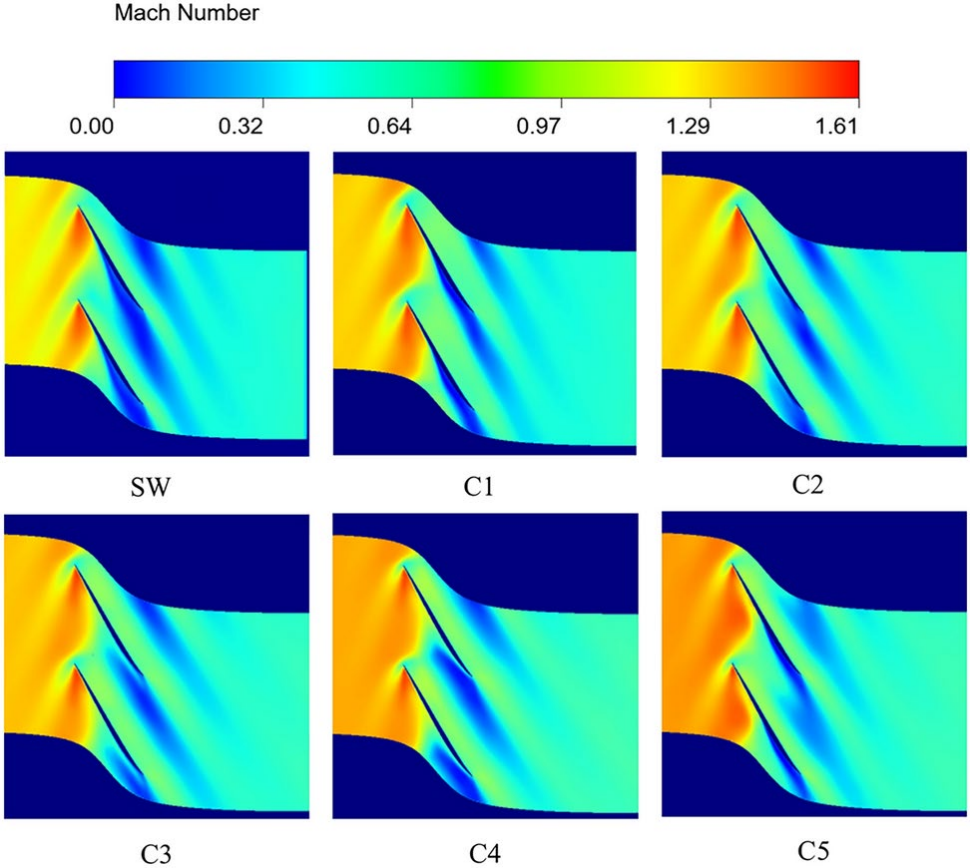
Prediction results of 95% span relative to Mach number near stall condition.

In order to verify the generalization of invisible conditions, the scalability and robustness of the model. Figure 19 shows the flow field prediction results of 95% blade height under near stall conditions after five kinds of casing treatments for Rotor37.

**Fig. 20:**
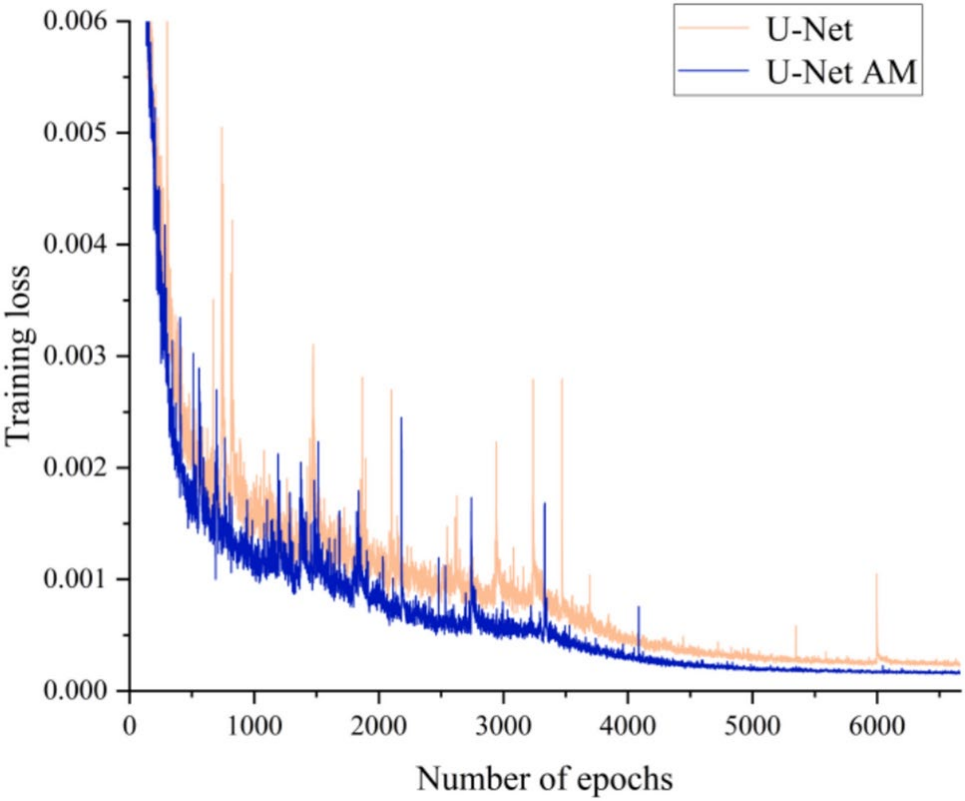
The training loss with and without the AM.

Figure 20 depicts the training loss evolution for prediction models, comparing those with and without the AM. Clearly, the model incorporating AM achieves rapid convergence within 3000 epochs and attains a significantly lower overall training loss. These results strongly indicate that integrating AMs enhance both the accuracy and training efficiency of prediction model, showcasing its effectiveness in improving predictive performance.

**Fig. 21:**
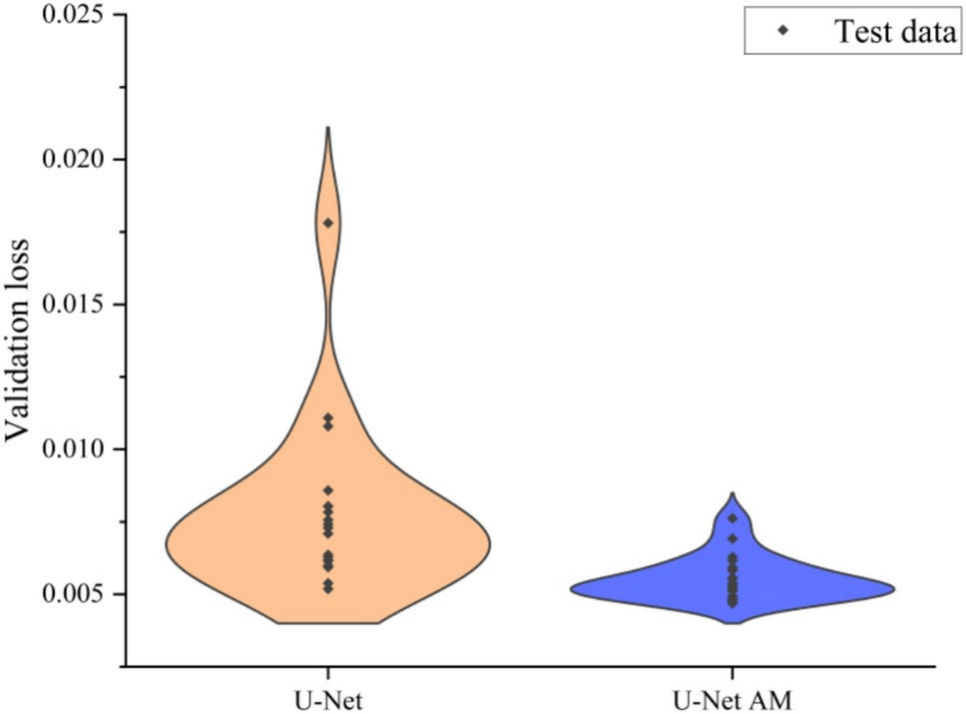
Distribution of validation loss with and without AM on the test dataset.

Figure 21 illustrates the distribution of mean absolute error across the test dataset. The shape of the violin represents the data distribution, with the thick black points shows where the data are located. The inclusion of AM results in a more concentrated distribution of validation loss. This improvement can be attributed to the enhanced capability of the networks to learn boundary features facilitated by CAM and SAM.

Table 5 shows the model parameters and GLFOPs of U-Net and U-Net AM, there is a slight increase in the GLFOPs when introducing the attention mechanism, but the overhead is negligible.

**Table 5:** Model parameters and GLFOPs.

Description	Parameters	GFLOPs
U-Net	2.92 million	39.11
U-Net AM	2.96 million	39.20

Table 6 presents the performance of the model in terms of prediction time and compares the time with a standard CFD solution. The reference time for the standard CFD method is obtained from the average of 50 random runs on a single core of the Intel Xeon Gold 6240 processor. The time required for the CNN model to make predictions is evaluated on the same CPU used to generate the real CFD data, as well as the Nvidia GeForce RTX-2080 Ti GPU. Since the training process can exhibit randomness due to factors such as the presence of potential local minima and non-deterministic GPU calculations, a single run may yield significantly different results. Therefore, the time results of 50 different runs are averaged using three different batch sizes (1, 10, and 50). The mean and standard deviation of running time, as well as the speedup values are shown. For all batch sizes used, the GPU-accelerated CNN model achieves a speedup of up to three orders of magnitude. Given that machine learning models can be efficiently run on GPUs, prediction times may vary depending on batch size, with the most efficient predictions observed at a batch size of 50.

**Table 6:** Run time and speedup comparisons.

Batch size	CFD(CPU)Time(s)	CNN model(GPU)Time(s)	Speedup
1	1.33 × 10^3^ ± 16.78	1.26 ± 3.12 × 10^−3^	1.06 × 10^3^
10	–	8.76 × 10^−1^ ± 3.12 × 10^−3^	1.52 × 10^3^
50	–	7.15 × 10^−1^ ± 3.12 × 10^−3^	1.86 × 10^3^

## Conclusion

In this work, a CNN based on U-Net architecture with attention mechanism is proposed to rapidly approximate the non-uniform steady flow of compressor blades. Through the formulations and examples, some concluding remarks can be drawn as follows:

1. Ground-truth CFD data are obtained by using a widely validated CFD solver and employed grayscale data processing to reduce data dimensionality, thereby reducing model training time.
2. Through extensive hyper-parameter search and evaluation of multiple architectures with various parameter configurations determined the optimal model and optimal learning rate for predicting the compressor internal flow field.
3. Unlike previous deep learning-based approximation methods, attention mechanisms are incorporated into learning framework to enhance the feature extraction effect of the network, especially when the flow exhibits rapid changes.
4. By comparing with the standard CFD method, the accuracy and performance of the model under three different working conditions are analyzed. Up to three orders of magnitude faster than the standard CFD method, while maintaining prediction errors below 1%.

Through the obtained results, the excellent performance suggests that it is an accurate and efficient way during aerodynamic design optimizations. The uncertainty in sparse and noisy data is recognized as an important factor that affects computational reliability. Future research is aimed at addressing the challenges posed by sparse and noisy data.

## Data Availability

The data used to support the findings of this study is available from the corresponding author upon request.

## References

[1] Chen, Y., Su, S. & Chen, Z. Compressor dynamic model and its parameter effects analysis for aeroengine active stability control. In *2018 37th Chinese Control Conference (CCC)*, 585–591 (IEEE 2018).

[2] Liu, H., Yue, S., Wang, Y. & Zhang, J. Unsteady study on the effects of matching characteristic of tandem cascade on the performance and flow at large angle of attack. *J. Therm. Sci.* **27**, 505–515 (2018).

[3] Zhang, L., Wang, S. & Zhu, W. Application of endwall contouring in a high-subsonic tandem cascade with endwall boundary layer suction. *Aerosp. Sci. Technol.* **84**, 245–256 (2019).

[4] Konrath, L., Peitsch, D. & Heinrich, A. An analysis of the secondary flow around a tandem blade under the presence of a tip gap in a high-speed linear compressor cascade. *J. Turbomach.* **144**, 101003 (2022).

[5] Rai, M. M. & Madavan, N. K. Aerodynamic design using neural networks. *AIAA J.* **38**, 173–182 (2000).

[6] Hu, L., Zhang, J., Xiang, Y. & Wang, W. Neural networks-based aerodynamic data modeling: A comprehensive review. *IEEE Access* **8**, 90805–90823 (2020).

[7] Luo, Q. et al. Effects of curved vanes on aerodynamic performance and flow structures in highly loaded tandem cascades. *Phys. Fluids* **36**, 036114 (2024).

[8] Zhang, B. et al. Study on flow separation and suction control of a compressor tandem cascade. *Proc. Inst. Mech. Eng. C J. Mech. Eng. Sci.* **237**, 5588–5601 (2023).

[9] Cao, P. et al. A study of the hydrodynamic characteristics of two-dimensional tandem cascades. *Water* **16**, 679 (2024).

[10] Swanson, R. & Langer, S. Steady-state laminar flow solutions for NACA 0012 airfoil. *Comput. Fluids* **126**, 102–128 (2016).

[11] Chen, D. et al. FlowDNN: A physics-informed deep neural network for fast and accurate flow prediction. *Front. Inf. Technol. Electron. Eng.* **23**, 207–219 (2022).

[12] Isola, P., Zhu, J.-Y., Zhou, T. & Efros, A. A. Image-to-image translation with conditional adversarial networks. In *Proceedings of the IEEE Conference on Computer Vision and Pattern Recognition*, 1125–1134 (2017)

[13] Krizhevsky, A., Sutskever, I. & Hinton, G. E. ImageNet classification with deep convolutional neural networks. *Commun. ACM* **60**, 84–90 (2017).

[14] Carrasquilla, J. & Melko, R. G. Machine learning phases of matter. *Nat. Phys.* **13**, 431–434 (2017). 10.1038/s41598-017-09098-0 PMC5562897 28821785

[15] Saetchnikov, I., Skakun, V. & Tcherniavskaia, E. Pattern recognition on aerospace images using deep neural networks. In *2020 IEEE 7th International Workshop on Metrology for AeroSpace (MetroAeroSpace)*, 336–340 (IEEE, 2020).

[16] Sofos, F., Drikakis, D., Kokkinakis, I. W. & Spottswood, S. M. Convolutional neural networks for compressible turbulent flow reconstruction. *Phys. Fluids* **35**, 116120 (2023).

[17] Zhang, Y., Sung, W. J. & Mavris, D. N. Application of convolutional neural network to predict airfoil lift coefficient. In *2018 AIAA/ASCE/AHS/ASC Structures, Structural Dynamics, and Materials Conference*, 1903 (2018).

[18] Yu, J. & Hesthaven, J. S. Flowfield reconstruction method using artificial neural network. *AIAA J.* **57**, 482–498 (2019).

[19] Mall, S. & Chakraverty, S. Comparison of artificial neural network architecture in solving ordinary differential equations. *Adv. Artif. Neural Syst.* **2013**, 181895 (2013).

[20] Yosifov, M. et al. Application of a modified 3D U-Net convolutional neural network architecture for the inspection of aerospace components. In *Proceedings of the 12th Conference on Industrial Computed Tomography (iCT)*, 1–6 (2022).

[21] Brunton, S. L., Noack, B. R. & Koumoutsakos, P. Machine learning for fluid mechanics. *Annu. Rev. Fluid Mech.* **52**, 477–508 (2020).

[22] Sofos, F., Drikakis, D., Kokkinakis, I. W. & Spottswood, S. M. Spatiotemporal super-resolution forecasting of high-speed turbulent flows. *Phys. Fluids* **37**, 016124 (2025).

[23] Sofos, F., Drikakis, D., Kokkinakis, I. W. & Spottswood, S. M. A deep learning super-resolution model for turbulent image upscaling and its application to shock wave-boundary layer interaction. *Phys. Fluids* **36**, 025117 (2024).

[24] Baert, L. et al. Aerodynamic optimization of the low-pressure turbine module: Exploiting surrogate models in a high-dimensional design space. *J. Turbomach.* **142**, 031005 (2020).

[25] Huang, A. C. et al. A variational multiscale method with discontinuous subscales for output-based adaptation of aerodynamic flows. In *AIAA Scitech 2020 Forum*, 1563 (2020).

[26] Kantor, E., Raveh, D. E. & Cavallaro, R. Nonlinear structural, nonlinear aerodynamic model for static aeroelastic problems. *AIAA J.* **57**, 2158–2170 (2019).

[27] Poulinakis, K., Drikakis, D., Kokkinakis, I. W., Spottswood, S. M. & Dbouk, T. Lstm reconstruction of turbulent pressure fluctuation signals. *Computation* **12**, 4 (2024).

[28] Milano, M. & Koumoutsakos, P. Neural network modeling for near wall turbulent flow. *J. Comput. Phys.* **182**, 1–26 (2002).

[29] Guo, X., Li, W. & Iorio, F. Convolutional neural networks for steady flow approximation. In *Proceedings of the 22nd ACM SIGKDD International Conference on Knowledge Discovery and Data Mining*, 481–490 (2016).

[30] Sarghini, F., de Felice, G. & Santini, S. Neural networks based subgrid scale modeling in large eddy simulations. *Comput. Fluids* **32**, 97–108 (2003).

[31] Thuerey, N., Weißenow, K., Prantl, L. & Hu, X. Deep learning methods for Reynolds-averaged Navier–Stokes simulations of airfoil flows. *AIAA J.* **58**, 25–36 (2020).

[32] Al-Shabili, A. H. & Selesnick, I. Positive sparse signal denoising: What does a cnn learn?. *IEEE Signal Process. Lett.* **29**, 912–916 (2022).

[33] Uhrig, J. et al. Sparsity invariant cnns. In *2017 International Conference on 3D Vision (3DV)*, 11–20 (IEEE, 2017).

[34] Qayyum, A., Malik, A., Saad, N. M. & Mazher, M. Designing deep CNN models based on sparse coding for aerial imagery: A deep-features reduction approach. *Eur. J. Remote Sens.* **52**, 221–239 (2019).

[35] Brajard, J., Carrassi, A., Bocquet, M. & Bertino, L. Combining data assimilation and machine learning to emulate a dynamical model from sparse and noisy observations: A case study with the Lorenz 96 model. *J. Comput. Sci.* **44**, 101171 (2020).

[36] Raissi, M., Perdikaris, P. & Karniadakis, G. E. Physics-informed neural networks: A deep learning framework for solving forward and inverse problems involving nonlinear partial differential equations. *J. Comput. Phys.* **378**, 686–707 (2019).

[37] Aliakbari, M., Mahmoudi, M., Vadasz, P. & Arzani, A. Predicting high-fidelity multiphysics data from low-fidelity fluid flow and transport solvers using physics-informed neural networks. *Int. J. Heat Fluid Flow* **96**, 109002 (2022).

[38] Cao, Z., Yao, W., Peng, W., Zhang, X. & Bao, K. Physics-informed mta-unet: Prediction of thermal stress and thermal deformation of satellites. *Aerospace* **9**, 603 (2022).

[39] Portal-Porras, K., Fernandez-Gamiz, U., Zulueta, E., Garcia-Fernandez, R. & Uralde-Guinea, X. CNN-based vane-type vortex generator modelling. *Eng. Appl. Comput. Fluid Mech.* **18**, 2300481 (2024).

[40] Wang, Y., Liu, T., Zhang, D. & Xie, Y. Dual-convolutional neural network based aerodynamic prediction and multi-objective optimization of a compact turbine rotor. *Aerosp. Sci. Technol.* **116**, 106869 (2021).

[41] Sun, D., Wang, Z., Qu, F. & Bai, J. A deep learning based prediction approach for the supercritical airfoil at transonic speeds. *Phys. Fluids* **33**, 086109 (2021).

[42] Geneva, N. & Zabaras, N. Quantifying model form uncertainty in Reynolds-averaged turbulence models with Bayesian deep neural networks. *J. Comput. Phys.* **383**, 125–147 (2019).

[43] Wang, R., Kashinath, K., Mustafa, M., Albert, A. & Yu, R. Towards physics-informed deep learning for turbulent flow prediction. In *Proceedings of the 26th ACM SIGKDD International Conference on Knowledge Discovery & Data Mining*, 1457–1466 (2020).

[44] Reid, L. & Moore, R. D. Design and overall performance of four highly loaded, high speed inlet stages for an advanced high-pressure-ratio core compressor (1978).

[45] Menter, F. R. et al. Ten years of industrial experience with the SST turbulence model. *Turbul. Heat Mass Transf* **4**, 625–632 (2003).

[46] Ronneberger, O., Fischer, P. & Brox, T. U-Net: Convolutional networks for biomedical image segmentation. In *International Conference on Medical Image Computing and Computer-Assisted Intervention*, 234–241 (Springer, 2015).

[47] de Ocáriz, S., Borde, H., Sondak, D. & Protopapas, P. Convolutional neural network models and interpretability for the anisotropic reynolds stress tensor in turbulent one-dimensional flows. *J. Turbul.* **23**, 1–28 (2022).

[48] Goodfellow, I., Bengio, Y., Courville, A. & Bengio, Y. *Deep Learning* (MIT Press, 2016).

[49] Ludwig, J. *Image Convolution* (Portland State University, 2013).

[50] Hu, J., Shen, L. & Sun, G. Squeeze-and-excitation networks. In *Proceedings of the IEEE Conference on Computer Vision and Pattern Recognition*, 7132–7141 (2018)

[51] Woo, S., Park, J., Lee, J.-Y. & Kweon, I. S. Cbam: Convolutional block attention module. In *Proceedings of the European Conference on Computer Vision (ECCV)*, 3–19 (2018)

